# WASP 8: The Next Generation in the 50-year Evolution of USEPA’s Water Quality Model

**DOI:** 10.3390/w12051398

**Published:** 2020

**Authors:** Tim Wool, Robert B. Ambrose, James L. Martin, Alex Comer

**Affiliations:** 1 Region 4, U. S. Environmental Protection Agency, Atlanta, GA 30303, USA; 2 Office of Research and Development, U.S. Environmental Protection Agency, Athens, GA 30605, USA; 3 Department of Civil and Environmental Engineering, Mississippi State University, Mississippi State, MS 39762, USA; 4 Platinum Code Solutions, LLC, Longmont, CO 80503, USA

**Keywords:** WASP, water quality, water quality model, environmental

## Abstract

The Water Quality Analysis Simulation Program (WASP) helps users interpret and predict water quality responses to natural phenomena and manmade pollution for various pollution management decisions. WASP is a dynamic compartment-modeling program for aquatic systems, including both the water column and the underlying benthos. WASP allows the user to investigate 1, 2 and 3 dimensional systems and a variety of pollutant types—including both conventional pollutants (e.g., dissolved oxygen, nutrients, phytoplankton, etc.) and toxic materials. WASP has capabilities of linking with hydrodynamic and watershed models which allows for multi-year analyses under varying meteorological and environmental conditions. WASP was originally developed by HydroScience, Inc. in 1970 and was later adapted by the US Environmental Protection Agency’s Large Lakes Research Station (LLRS) for applications to the Great Lakes. The LLRS first publicly released the model in 1981. WASP has undergone continuous development since that time and this year will mark its 50th anniversary. This paper follows the development of WASP from its origin to the latest release of the model in 2020, documenting its evolution and present structure and capabilities.

## 1. Introduction

The first public domain release of the Water Quality Analysis Simulation Program (WASP) by the United States (US) Environmental Protection Agency (USEPA) Environmental Research Laboratory-Duluth (ERL-D) Large Lakes Research Station [1], set out its guiding philosophy and approach:

“The application of mathematical modeling techniques to water quality problems has proved to be a powerful tool in water resource management. As a diagnostic tool, it permits the abstraction of a highly complex real world. Realizing that one can never fully expect to detail all the physical phenomena that comprise our natural world, one attempts to identify and include only the phenomena, be they natural or man-made that are relevant to the water quality problem under consideration. As a predictive tool, mathematical modeling permits the forecasting and evaluation of the effects of changes in the surrounding environment on water quality. Although engineering insight and political and socio-economic concerns play important roles in water resource management, some water quality problems are of such a highly complex nature that the predictive capability of mathematical models provides the only real means for screening the myriad number of management alternatives.”

This is just as true today as in 1981. Prior to, and since that original release, a large number of other models intended to predict water quality of surface waters have been developed, most designed for a particular waterbody type and/or water quality issue. While relatively few extant models can demonstrate a continuous record of model development and application over a fifty-year period, this year (2020) marks the 50th anniversary of WASP.

The WASP model was originally developed by HydroScience in 1970 and released in the public domain in 1981 [1]. As with a number of models in use today, WASP was originally developed and used in the private sector. The model was applied by HydroScience and Manhattan College during the course of a number of projects for the Great Lakes for the USEPA ERL-D’s Large Lakes Research Station (LLRS) at Grosse Ile, MI. That lab (under the direction of Bill Richardson) recognized the utility of WASP, leading to its public release in 1981 [1]. WASP has remained under continuous development by the USEPA since that time. That WASP has remained in continuous use can be attributed in large part to its original generalized theoretical and computational design and modular program logic. This allowed its application to dynamic (time-variable) prediction of a wide variety of water quality constituents over a variety of spatial and temporal scales.

WASP is a differential, spatially resolved, mass balance, fate and transport modeling framework structured to allow users to simulate concentrations of environmental contaminants in surface waters and bottom sediments. WASP has been applied to a range of different surface water systems, addressing a range of environmental contaminants. Some published applications of WASP in the U.S. include: nutrient loading on water quality in Tampa Bay, Florida [2]; mercury fate and transport in the Carson River, Nevada [3]; a total maximum daily load analysis (TMDL) for nutrients in the Neuse River Estuary, North Carolina [4]; mercury remediation strategies in the Sudbury River, Massachusetts [5]; the fate and transport of multi-walled carbon nanotubes in Brier Creek, Georgia [6]; and, phytoplankton in St. Louis Bay, Mississippi [7]. Some published applications of WASP outside the U.S. include: impacts of climate change on water quality of Chungju Lake (South Korea) [8]; dissolved oxygen in the Danshui and Chungkang Rivers, Taiwan [9]; algal dynamics in an urban river in Beijing [10]; dissolved oxygen depletion in diverted floodwaters of the Elbe River, Germany [11]; eutrophication control in the Keban Dam Reservoir, Turkey [12]; and mercury exports from the Marano–Grado Lagoon to the Adriatic Sea, Italy [13]. Numerous applications of WASP published in the peer reviewed literature are compiled in a recent U.S. EPA report [14].

The general design framework of WASP and its flexibility has allowed the model to evolve over the past fifty years from its initial development into one of the most widely used dynamic models throughout the US and the world. The purpose of this paper is to describe that evolution that has led to the model as it is structured today. That evolution has resulted in part due to the rapid changes that have occurred in computer technology, as well as the changing needs for making environmental decisions. Despite the many significant enhancements over this period, WASP remains firmly based on a model framework that in many ways has remained unchanged from the original model developed 1970 and first released to the public domain in 1981 [1].

### 1.1. General Framework

**Figure 1.**
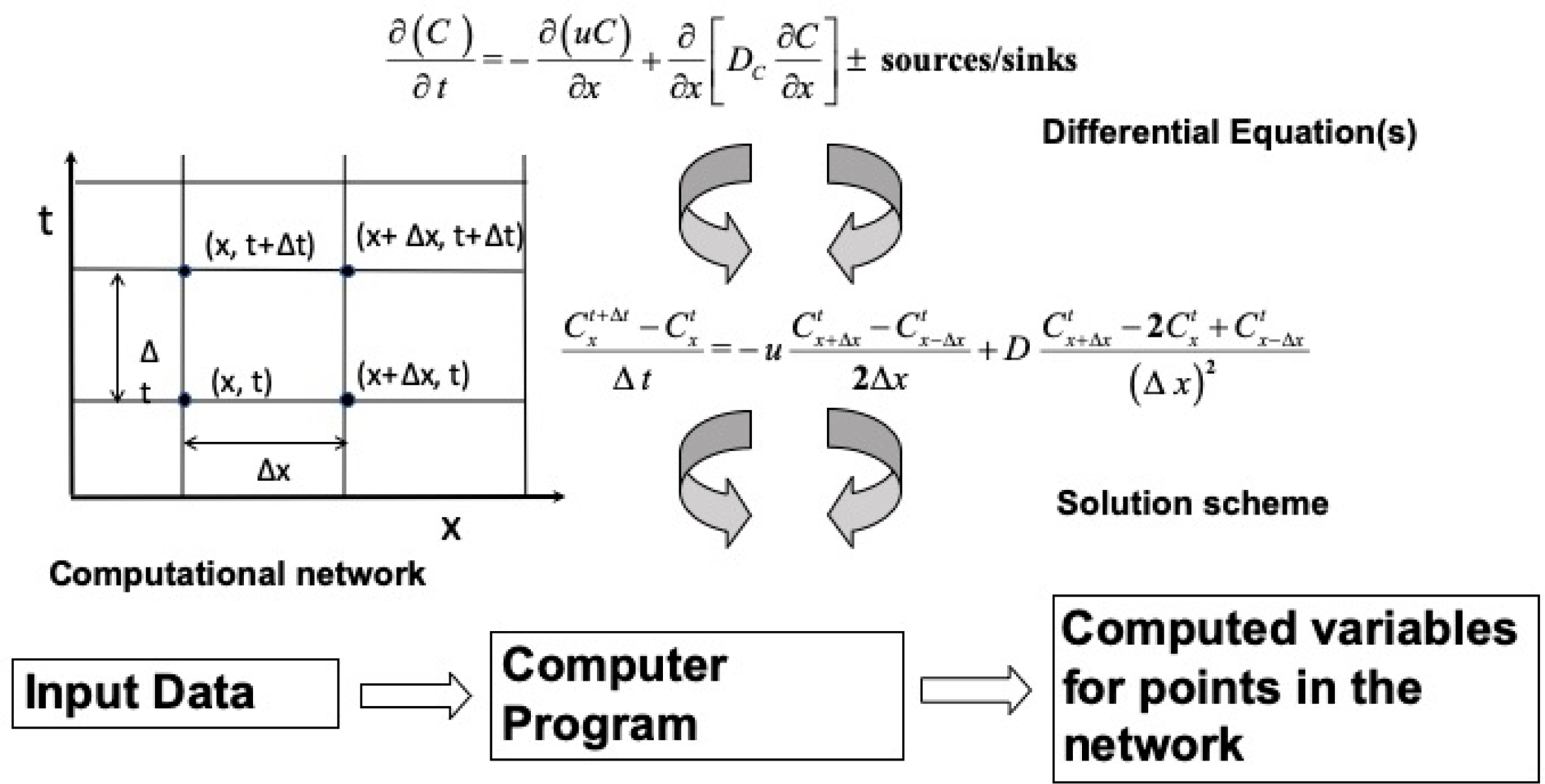
Example of model development process for a 1-dimensional finite difference model.

The general framework for any model development (Figure 1) consists of first developing theoretical equations, typically in the form of differential equations. For most practical situations, these equations cannot be solved analytically so that some numerical representation is required. That representation along with a selected computational network leads to the development of numerical solutions. The source sink terms are specific to a particular chemical (of concentration C) and include external loads, boundary forcing and kinetics associated with chemical and biologic processes. Once the general theoretical and numerical frameworks are selected along with the degree of kinetic complexity, all of this must be programmed into some computer code. That code must also include methods for getting information into the code and output from the predictions. Each of these steps requires a large number of decisions, all of which can affect the usability and applicability of what then has become a computer model.

### 1.2. General Mass Balance Equation

The theoretical foundation of the WASP model is the conservation of mass. A mass balance for some arbitrary water quality constituent with concentration C, over a control volume of arbitrary size in Cartesian coordinates results in the three-dimensional advection-diffusion equation a form of which is illustrated below

(1) $$\frac{\partial C}{\partial t}=-\frac{\partial U_{x}C}{\partial x}+\frac{\partial }{\partial x}\left(E_{x}\frac{\partial C}{\partial x}\right)-\frac{\partial U_{y}C}{\partial y}+\frac{\partial }{\partial y}\left(E_{y}\frac{\partial C}{\partial y}\right)-\frac{\partial U_{z}C}{\partial z}+\frac{\partial }{\partial z}\left(E_{z}\frac{\partial C}{\partial z}\right)+S_{L}+S_{B}+S_{K}$$

where:

C = concentration of the water quality constituent, M/L^3^

t = time, T

U_x_, U_y_, U_z_ = longitudinal, lateral and vertical advective velocities, L/T

E_x_, E_y_, E_z_ = longitudinal, lateral and vertical diffusion coefficients, L^2^/T

S_L_ = direct and diffuse loading rate, M/L^3^ T

S_B_ = boundary loading rate (including upstream, downstream, benthic and atmospheric), M/L^3^ T

S_K_ = total kinetic transformation rate; positive is source, negative is sink, M/L^3^ T

The basic concept of writing a mass balance equation for a body of water is to account for all of the material entering and leaving the water body via direct addition of material (runoff and loads), via advective and dispersive transport mechanisms and via physical, chemical and biologic transformations [1].

### 1.3. Computational Network

Virtually all mechanistic water quality models are based on the above equation, which for all practical purposes must be solved numerically. The numeric solution first starts with the selection of a numeric method to represent the equation (e.g., finite difference, finite element, etc.) and computational grid. For WASP, the mass balance equation is represented using finite differences.

Some models solve the mass balance equation in its three-dimensional form, while others integrate the model over space and are two- or one-dimensional, resulting in models that can only be applied in one, two or three dimensions. The approach used in WASP is to integrate the equation over a completely mixed finite segment or “box” which may also be referred to as an integrated control volume as illustrated below.

(2) $$\frac{\Delta \left(V_{i}C_{i}\right)}{\Delta t}=\sum_{j}Q_{j,i}C_{j}-\sum_{k}Q_{i,k}C_{i}+\sum_{j}\frac{E_{i,j}A_{i,j}}{L_{i,j}}\left(C_{j}-C_{i}\right)+S_{L,i}+S_{B,i}+S_{K,i}$$

where *i* and *j* are pointers to either a boundary or adjoining segments; *Q* = advective flowrate (L^3^/T), *E* = dispersion coefficient (L^2^/T) across an interface, *A* = interfacial area (L^2^) and L = the characteristic length (L).

This general “box” model formulation allows the completely mixed finite volumes or segments to be configured in arbitrary numbers and arrangements. For example, segments may be “stacked” or shaped to form one, two- or three-dimensional networks. This allows WASP models to be applied to networks composed of one to thousands of segments arranged in one to three dimensions as dictated by the physics of the system being modeled (e.g., to rivers, streams, lakes, reservoirs, estuaries, etc.), variations in water quality and regulatory point of compliance (e.g., at some depth or time).

The general flexibility of the framework is one major factor that has contributed to the wide use and longevity of the WASP model. That flexibility is included in user input as described below allowing users to configure WASP as needed for their particular problems. That generalization is also carried through from the numerical framework to the transport and kinetic formulations. The general philosophy guiding WASP developers has always been to keep WASP a general-purpose computer program. WASP is not considered a model until it is configured by the user for a particular application. As a consequence, the WASP program structure is designed to minimize the number of choices made by the developers and to allow the decisions on input to be made by the model users, while providing a generalized state of the art platform on which they can build their model.

### 1.4. WASP Program Logic

The general program logic for WASP has remained relatively unchanged over the past 50 years, although the specific implementation has greatly improved. The general programming logic may be illustrated using the numerical form of the mass balance equation:

(3) $$[\underset{4}{\underset{︸}{\frac{\Delta \left(V_{i}C_{i}\right)}{\Delta t}}}]=\left[\underset{1}{\underset{︸}{\sum_{j}Q_{j,i}C_{j}-\sum_{k}Q_{i,k}C_{i}+\sum_{j}\frac{E_{i,j}A_{i,j}}{L_{i,j}}\left(C_{j}-C_{i}\right)}}\right]\;\;+\left[\underset{2}{\underset{︸}{S_{L,i}+S_{B,i}}}\right]+\left[\underset{3}{\underset{︸}{S_{K,i}}}\right]$$

where (1) refers to advective and dispersive transport, (2) external loadings and (3) total kinetic transformations, which are equated to the rate of change in constituent mass (4).

### 1.5. Transport

The basic solution to the above equation is to start with specified initial conditions (e.g., volume, initial concentration, etc.) for each segment and water quality constituent (state variable) and then compute new volumes and concentrations at the end of the specified time increment. This set of operations is repeated over a specified period of time for which relevant transport terms, loads, boundary conditions and other factors are specified.

Time varying flows, rates of dispersion, loads and boundary conditions must be specified by the user. For complex water bodies, the transport information can be predicted by external hydraulic or hydrodynamic models which are linked to WASP using input (linkage) files. The hydraulic and hydrodynamic models which have been linked with WASP have also evolved during the past 50 years which will be described in the review of WASP’s evolution.

The number of state variables varies with the type of model (e.g., eutrophication, toxics, etc.). The user must also input the relevant kinetic rate constants, coefficients and other information necessary to compute the changes in concentration due to transformations.

**Figure 2.**
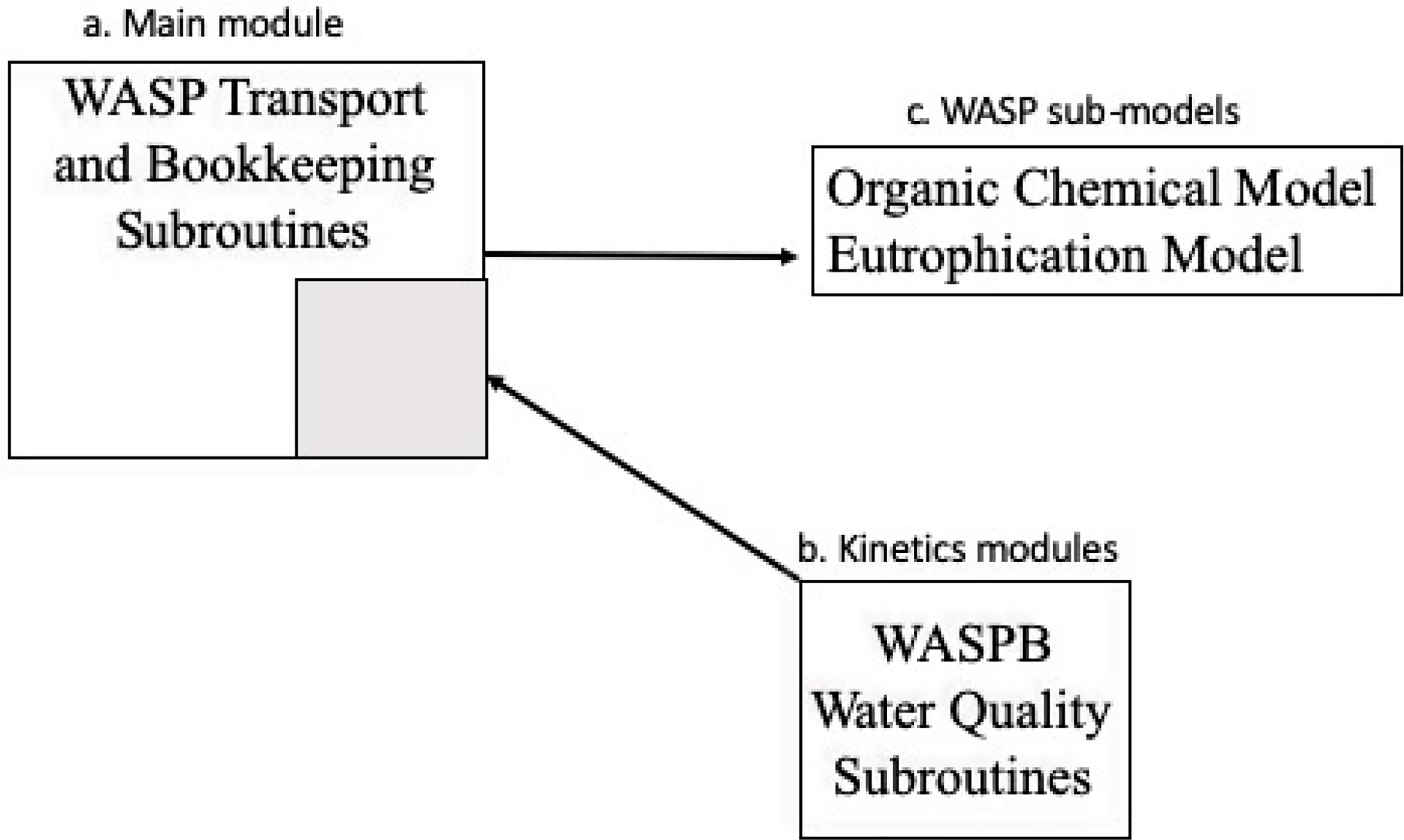
Water Quality Analysis Simulation Program (WASP) structure: selected kinetics modules (**b**) are linked with the main module (**a**) to produce problem-specific submodels (**c**).

This general WASP program structure (Figure 2) consists of main module that does all of the bookkeeping and transport coupled with a kinetic module to produce a problem-specific submodel. The main module is the same regardless of the kinetic module. This structure allows model networks to be used for all WASP submodels.

The main WASP module provides subroutines to read input and then compute the mass changes (M/L^3^ T) due to the transport, loads and boundary conditions which are independent of the specific state variables being simulated. The result is a block of code that performs these operations and the bookkeeping associated with the specified number of segments and state variables. The same code is used for any model to which WASP is applied. In the original WASP (and still today) the input subroutines are numbered WASP1 through WASP11. WASP12 computes the time variable computations. The transport routine also computes (or inputs) the time variable segment volumes.

Associations of input and dynamic computations of specific state variables to specific water quality constituents (e.g., dissolved oxygen, etc.) are accomplished in the kinetic module. The kinetic formulations specific to a particular water quality submodel are included in a separate generic subroutine, WASPB (along with associated subroutines and functions written to support the water quality kinetics). These subroutines compute the kinetic changes for each state variable for each reaction process and then pass the resulting kinetic derivatives (M/L^3^ T) back to the main transport code. At the end of each time step, the changes in kinetics, volumes, transport and loadings are used to compute new segment concentrations for each of the state variables simulated. A wide variety of WASP routines have been developed and applied over the past 50 years.

### 1.6. Numerical Solution

Since its inception, WASP has used an explicit finite difference Euler technique to solve Equation (3). Spatially, WASP uses a backward difference approximation for the advection derivative and a central difference approximation for the dispersion derivative. The kinetic derivative is calculated at the midpoint of each segment. WASP sums these components of the mass derivatives for every segment during every time step between initial time and final simulation time. Given concentrations and volumes for all segments at time *t*, WASP calculates new masses at *t* + Δ*t* using a one-step Euler scheme. Given new constituent masses at time *t* + Δ*t*, WASP divides by the new volumes to obtain the new concentrations.

Originally the user had to specify the time step(s) to be used. The model time step is now calculated internally based on flow and kinetic constraints, subject to user specified maximum and minimum values and time step multiplier. Typically, values range between 0.0001 and 0.5 days.

### 1.7. External Linkages

WASP was originally developed as a standalone program that can receive information from or provide information to other models as dictated by the needs of a particular application. Most linkages typically require that the external models be configured to write out information that WASP can use or read information from WASP in a specified format.

During its evolution, WASP has been coupled with a wide variety of external models. These have included hydraulic, hydrodynamic and hydrologic models, watershed loading models, food chain models and others. While WASP has used the predictions from some models and provided predictions to other models, they have in general not been directly incorporated into the WASP structure. As a result, the WASP model has not become dependent upon the parallel development of models such as those for predictions of hydraulics and hydrology. Many of the models with which WASP has been coupled have come and gone, with few standing the test of time. In some cases, particularly useful models have been distributed with WASP and while this separation may not be transparent to the user, the separation has in general been maintained and has contributed to the longevity of WASP.

### 1.8. WASP Input/Output Structure

**Figure 3.**
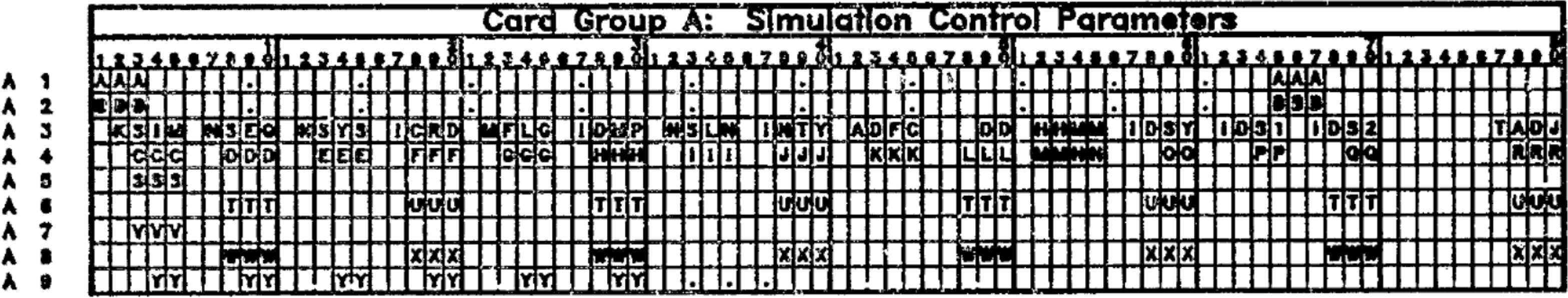
Early card image input for WASP.

The general structure of WASP includes a main module that does all of the bookkeeping (and input, output, etc.) and transport (advective and dispersive) coupled with a kinetic module (WASPB). The input to WASP has remained essentially the same over the years, but the manner in which the user provides these inputs has varied considerably. Originally, the user input was developed in Card Groups where during the early development of WASP model computer input was via “punch” cards (Figure 3). The WASP input Card groups included:

- Model Identification and System Bypass Options
- Exchange Coefficients
- Segment Volumes
- Flow
- Boundary Conditions
- Forcing Functions
- Parameters
- Constants
- Miscellaneous Time Functions
- Initial Conditions for each system of the model

One of the powerful generalized features of WASP is the liberal use of scale and conversion factors along with bypass options throughout the model. These features were included in the initial release [1] and retained and expanded upon in later versions. Most input variables had their own respective scale and conversion factors which were useful if the user wanted to keep input in their native units. These scale and conversion factors also proved very useful in model testing and applications. For example, if a user wanted to determine the impact of doubling a time variable boundary condition, they could do so by simply changing the scale or conversion factor rather than modifying the input. The bypass options were also utilized throughout the input and allowed users to turn input “on” or “off” (bypass or simulate). For example, for earlier WASP kinetic configurations with a constant number of state variables, the user could simply bypass or turn off those state variables they did not want to simulate. This provides a considerable flexibility for the user in configuring kinetics most applicable for their particular situation. The user can also turn “on or off” transport for a particular state variable (advective or dispersive or both). Bypasses are also provided for a wide variety of other input (now they are simply checked (on) or unchecked (off)). This feature provides considerable flexibility and has proved extremely useful in model evaluations and applications.

Transport information can be provided to the model as specified by the user or read from linkage files for hydraulic or hydrodynamic models. These models may be external to the WASP modeling system or included with WASP distribution, such as some of the stream models in the present model. Forcing may include state variable specific boundary conditions associated with a specific flow or direct loadings. Kinetic constants and coefficients are also associated with each state variable as are initial conditions for each state variable and model segment.

The WASP model also allows input of parameters which may be varied by segment and include things some as wind speed, air temperature and other factors. These parameters are variables on which predictions depend (for the specific kinetic model) but are not predicted by the models. Time functions are also available for some parameters providing for their variation with time.

## 2. WASP: The First 50 Years

### 2.1. WASP: The Origin (1970–1983)

**Figure 4.**
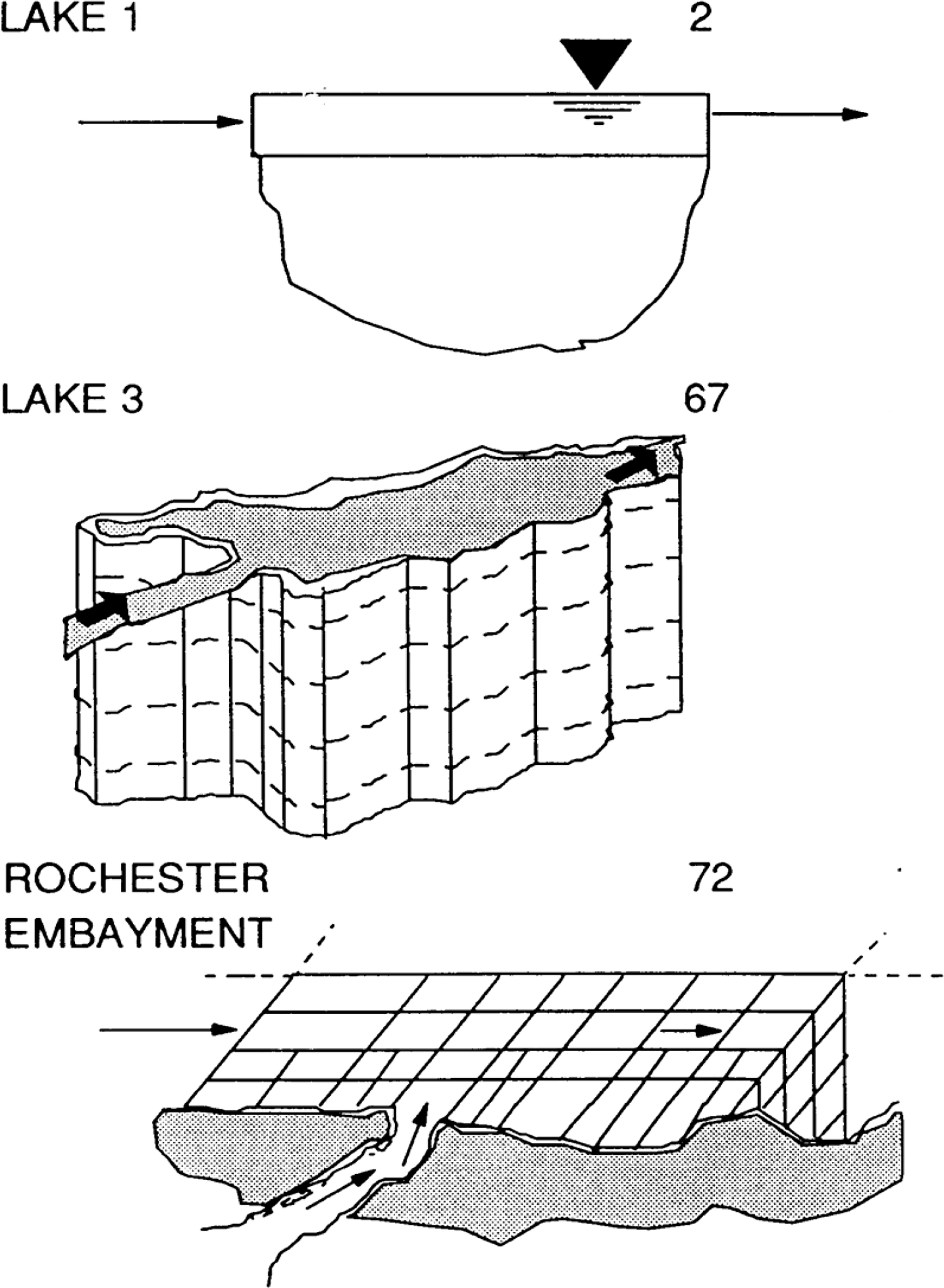
WASP Segmentation for Lake Ontario (with number of segments indicated).

The origin of WASP can be traced to Hydro Science in 1970 and the pioneering work by Dominic M. Di Toro, James J. Fitzpatrick, John L. Mancini, Donald J. O’Conner, Robert V. Thomann and others. Early applications included modeling phytoplankton dynamics in the Sacramento–San Joaquin Bay Delta [15] and the Western Delta–Suisun Bay [16]. Over the next decade the model was applied to a variety of waterbodies in 1–3 dimensional applications to both conventional and toxic materials. Of particular relevance were applications to the Great Lakes supported by the USEPA ERL-D Large Lakes Research Station (LLRS). These included a two layer eutrophication model of Lake Ontario [17,18] (LAKE1), a three dimensional eutrophication model of Lake Ontario (LAKE3) and a three dimensional model of Rochester Embayment [19] (all shown in Figure 4), plus applications to Lake Huron and Saginaw Bay [20] and Lake Erie [21]. These applications led to recognition of the merits of the model structure and the public domain release of the then named Water Quality Analysis Simulation Program (WASP) by the LLRS in 1981 [1].

#### 2.1.1. Water Quality/Regulatory Environment

When WASP was developed, the continuing eutrophication of the Great Lakes was a matter of international concern. A number of efforts were underway by the U.S. and Canada to assess the extent and impacts of eutrophication and international agreements developed for nutrient reduction (e.g., the Great Lakes Water Quality Agreement of 1972 between the U.S. and Canada which focused on phosphorus loadings). To assist in the effort, a number of empirical and mechanistic models were developed for the express purpose of relating nutrient loads to impacts. A number of these studies were supported by the LLRS and based on the HydroScience and Manhattan College versions of WASP ultimately leading to its adaption and use. This was also a time of proliferation of models of varying kinetic complexity, so that here was no single kinetic construct that was widely accepted.

#### 2.1.2. Programming Environment

WASP was written in FORTRAN IV using a modular, subroutine-oriented program structure. WASP was applied at the LLRS, Manhattan College and at the Environmental Research Lab-Athens on PDP-11 minicomputers which were developed by Digital Equipment Corporation (DEC) beginning in 1970. Almost unimaginable today, the core capacity of these computers was 32 KB (Kilobytes, 103 bytes), so that a lot of code involved swapping information in and out of memory. The number of state variables and model segments were specified in a file used to allocate memory during compilation and they ranged from a maximum of 16 state variables (programming systems) and 40 segments [1]. Guides were provided in the user documentation on how to change the dimensions of arrays where necessary.

#### 2.1.3. User Interface (Input/Output)

Data input to WASP was provided on punched cards and, in later versions, ASCII files with card image format. Each Card Group (A, B, etc.; Figure 3) had specific input associated with it. This input structure was preserved in WASP input files over the next several decades. Two card groups were provided for specifying a print interval (the total number of printouts had to be less than 41), the system (state variable) and segment combinations to be output (maximum of 8) and display variables.

#### 2.1.4. Kinetic Structure

The original public release of WASP in 1981 [1] did not include a water quality model. Instead, WASP was written to allow users to develop their own water quality routines and couple them with WASP (through addition of a WASPB module). The target audience for the user’s manual was the system analyst, whose responsibility it would be to design, develop and debug new kinetic models for end users, as well as the end user who must prepare the data input to the program. The user manual described the program structure and provided instructions and examples on how to develop and couple water quality modules with WASP. The manual also provided explicit instructions on the design and testing of those modules and example coding.

#### 2.1.5. Auxiliary/Support Programs

WASP was distributed with a companion program, the Model Verification Program (MVP) which computed statistics to aid in assessing model predictions.

#### 2.1.6. Model Linkages

WASP did not compute hydrodynamics. As a result, the transport mechanisms, both advective and dispersive, had to be specified by the user. While WASP was essentially a standalone program, i.e., not executed in conjunction with another program (that may produce for example hydrodynamics or runoff waste loads), the user’s manual [1] included logic for it to be user-programmed to interface with other computer programs if needed. An example was also described whereby WASP was modified to accept time-variable non-point runoff flows and waste loads from a catchment rainfall runoff model.

### 2.2. WASTOX and TOXIWASP (1983, 1984)

#### 2.2.1. Water Quality/Regulatory Environment

During this period, a major shift occurred in regulatory emphasis from conventional to toxic materials. The Resource Conservation and Recovery Act (RCRA) governing the disposal of solid and hazardous waste was passed in 1976. The Superfund Act (Comprehensive Environmental Response, Compensation and Liability Act or CERCLA) was enacted in 1980. Contamination in the Great Lakes and elsewhere became international issues, with the primary contaminates including pesticides and hydrophobic organic toxicants such as PCBS, Dioxins, PAH’s and VOC’s. Both point and non-point sources were of issue (e.g., the Great Lakes Water Quality Agreement of 1978 between the U.S. and Canada which focused on persistent toxicants, [22–24]).

During this period, TOXIWASP was developed at the ERL-Athens Center for Water Quality Modeling (CWQM, [25]). TOXIWASP combined most of the kinetic structure of EXAMS-2 described by Burns et al. [26] with the transport capabilities of WASP. The Exposure Analysis Modeling System (EXAMS) first published in 1982 [26] was developed for the rapid evaluation of the fate, transport and exposure concentrations of synthetic organic chemicals and that capability was incorporated into WASP resulting in TOXIWASP. A similar model developed during this period was WASTOX. The WASTOX chemical model was based on a toxicant model coupled with WASP [27], but also included a companion food chain model [28]. The hydrophobic contaminants of concern during this period, and today, accumulated in sediments and bioaccumulated through the food chain so that one of the questions often posed was “when can I eat the fish”?

#### 2.2.2. Programming Environment

**Figure 5.**
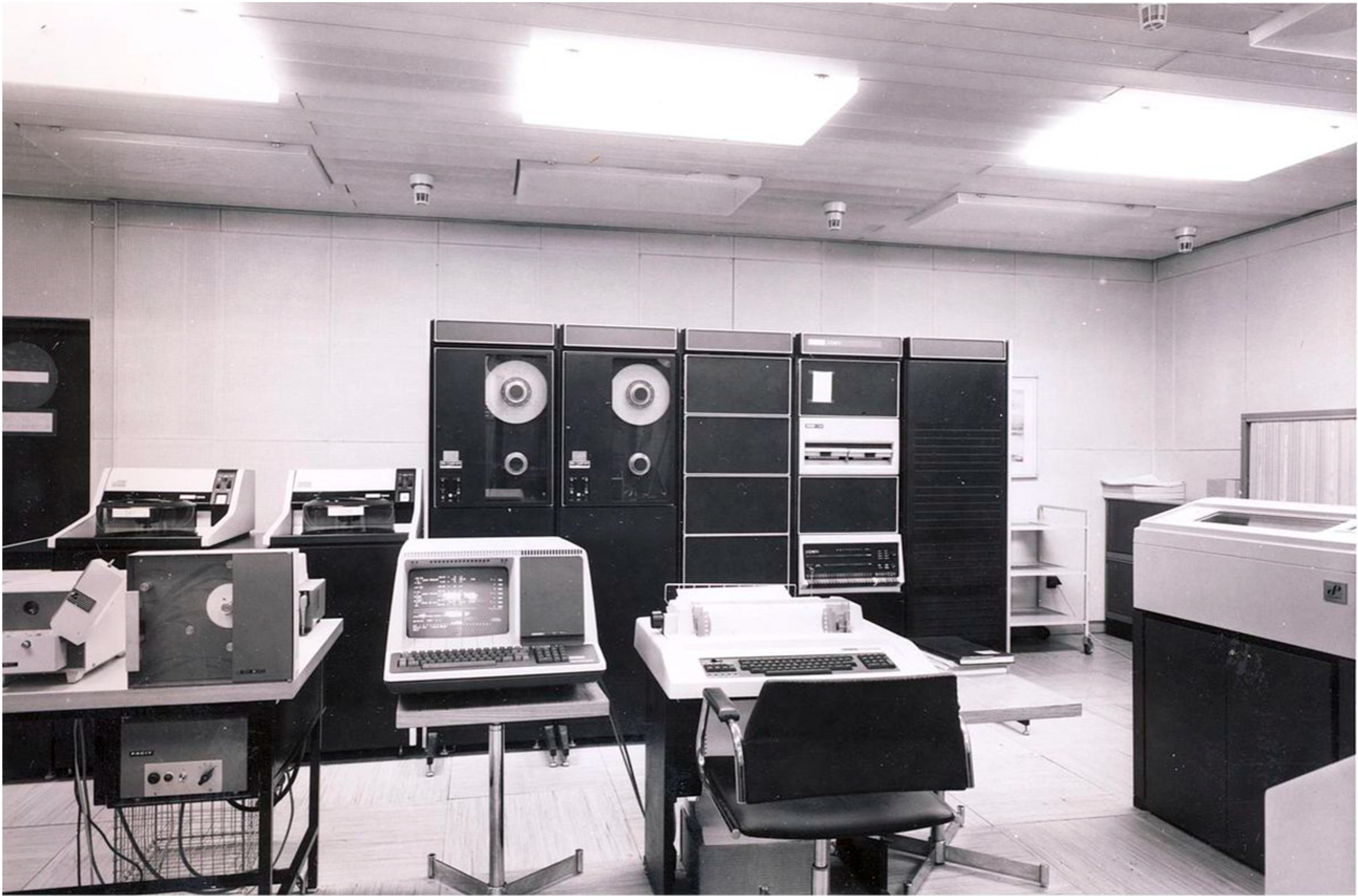
PDP 11/70.

TOXIWASP was also written in FORTRAN IV and was designed for an IBM 370 (OS/MVS Operating System) or a PDP 11/70 computer system (IAS operating system) that operate in a 64 Kilobyte’s or memory user area. These minicomputers were basically mini mainframe computers that were shared among multiple users. The original IBM 370 minicomputer was announced in 1970. The PDP-11 (Figure 5) was a series of 16-bit minicomputers sold by Digital Equipment Corporation (DEC) from 1970 into the 1990 s. The number of segments was specified as a model parameter in WASP and two configurations were released, one capable of simulating 50 segments and the other 100 segments.

#### 2.2.3. User Interface (Input/Output)

As with the original WASP, input was in a series of card image formats input via a cathode ray tube (CRT) or card deck (Figure 3).

#### 2.2.4. Kinetic Structure

**Figure 6.**
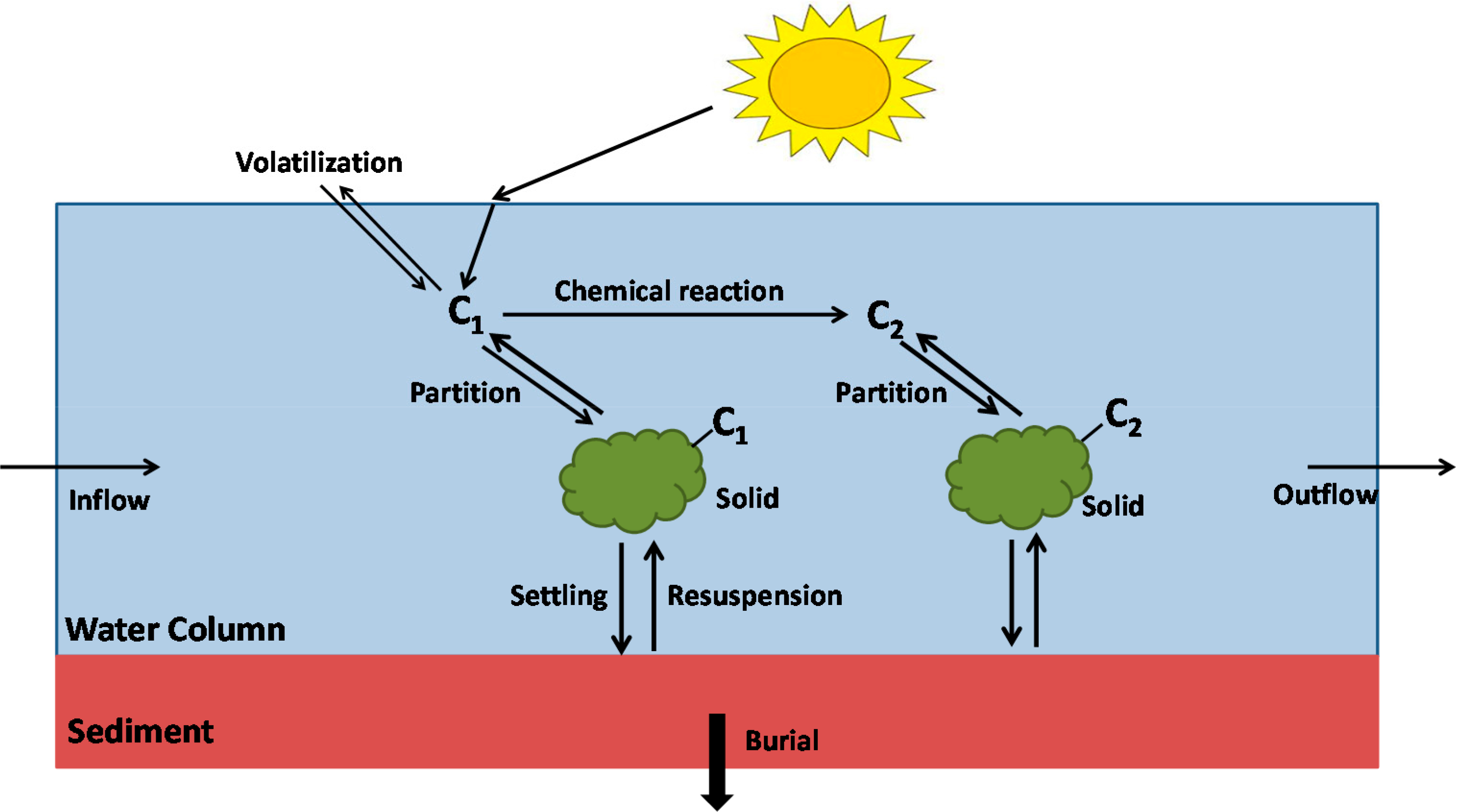
Framework and selected processes for TOXIWASP (chemical 1); later TOXI modules provided for up to three chemicals.

The kinetic structure included kinetic structure included one arbitrary contaminant and total solids in both the water column and sediments. Transformation and degradation processes such as sorption, volatilization, photolysis, oxidation, biodegradation and hydrolysis were included. One major change from most previous WASP models was the inclusion of both water column and sediment segmentation and descriptive transport (e.g., resuspension, sedimentation, burial) for the sediments and associated contaminants between the bed and water column as illustrated in Figure 6. Previously, only the water column was simulated and the impacts of the sediment bed specified (e.g., sediment oxygen demand).

#### 2.2.5. Auxiliary/Support Programs

No auxiliary programs were provided with TOXIWASP.

#### 2.2.6. Model Linkage

Perhaps the first linkage of WASP to a hydrodynamic model occurred with the linkage of DYNHYD with TOXIWASP in an application to the Delaware River by Ambrose [29,30]. These models were applied to calculate the upstream migration of seven volatile organic chemicals from a wastewater effluent to a drinking-water treatment plant in Philadelphia PA requiring simulation of hydrodynamics and mass transport; sediment transport; and, chemical transport and transformation.

### 2.3. WASP3 (1986), WASP4 (1988), WASP5 (1993)

Initially during this period model development was supported by the U.S. EPA, Center for Water Quality Modeling (CWQM), Athens, GA which later became the Center for Exposure Assessment Modeling (CEAM). CEAM expanded the range of models developed, provided support in their application and training in their use.

WASP3 was developed to integrate models of toxicant kinetics with an eutrophication model, providing the capability of simulating toxicants and eutrophication within a single model framework. WASP4 represented a restructuring and updating of the code, in particular the toxicant model. WASP5 included better transport linkages and initial versions of pre- and post-processors (W5DSPLY).

#### 2.3.1. Water Quality/Regulatory Environment

During this period both contamination by toxicants, such as persistent organic pollutants (POPs) and others, as well as eutrophication remained critical environmental issues to which models were applied. Of particular note during this period was the emergence of TMDL’s. During the early years of the implementation of the Clean Water Act (CWA), point sources were the major focus for regulation and hence model applications (e.g., the development of waste load allocations). Waste load allocation models then typically considered only the point sources and receiving water. However, following that implementation of point source controls, a large percentage of U.S. waterbodies were still not meeting water quality criteria. Under the CWA (303(d)), those waterbodies must then become the focus of TMDLs, which drove the need to include both point and nonpoint sources in model applications.

#### 2.3.2. Programming Environment

**Figure 7.**
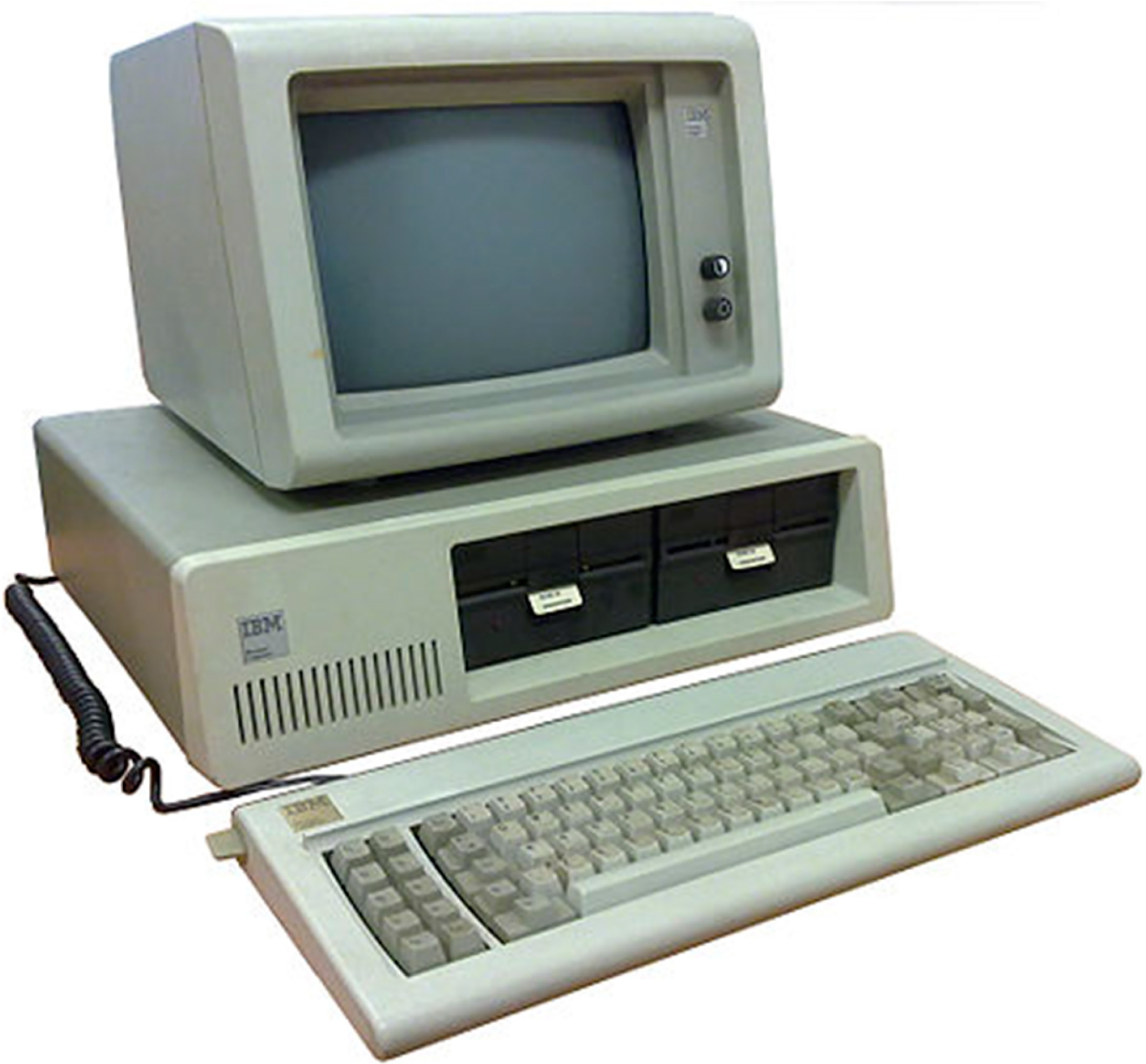
IBM 5150 PC with IBM 5151 monitor.

During this phase of WASP development, microcomputers became available and could be located on an individual’s desktop. Computer access was greatly improved. The IBM Personal Computer (IBM 5150) was introduced in August 1981 (Figure 7) and the operating environment was the Disk Operating System (DOS). Multiple 32 K and 64 K memory cards could be plugged into the option slots to increase memory to 256 K, which was a significant improvement over what was available previously. Microsoft introduced the Windows operating environment in 1985 as a graphical operating system shell for MS-DOS. Windows came to dominate the world’s personal computer (PC) market by 1984. This began the era of migrating WASP from a mainframe/minicomputer to the Windows PC environment.

#### 2.3.3. User Interface (Input/Output)

Initial efforts of developing IBM PC based graphical user interfaces for WASP began with the development of pre- and post-processors which were distributed with WASP5. These included PREDYN, W5DSPLY and PLOT. PREDYN was an interactive preprocessor program for DYNHYD. W5DSPLY was a tabular post processor program for TOXI, EUTRO and DYNHYD. PLOT was a graphical post processor for TOXI, EUTRO and DYNHYD.

#### 2.3.4. Kinetic Structure

**Figure 8.**
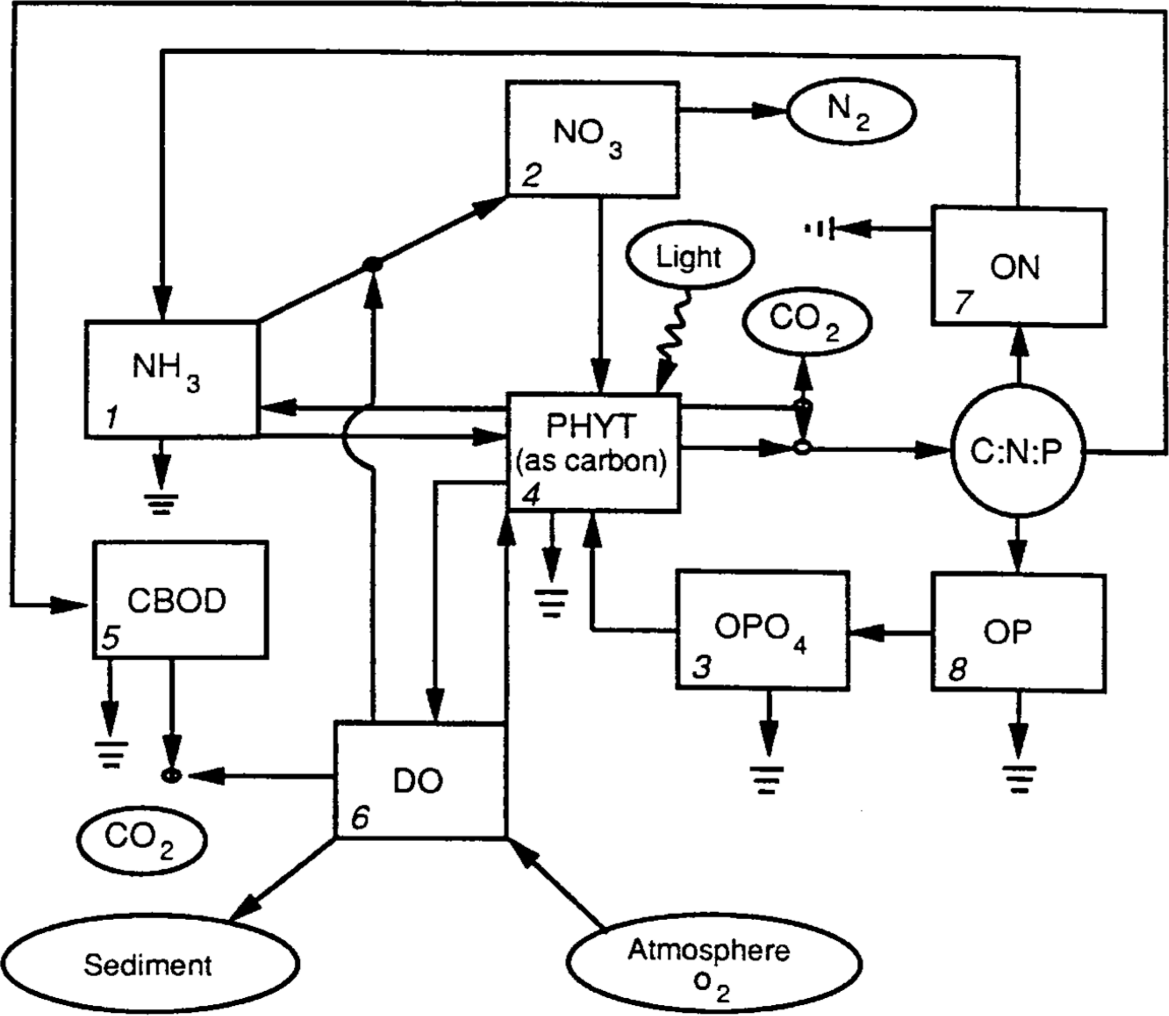
Eutrophication kinetic model for WASP versions 3–6.

The WASP3 release included both a toxicant model (TOXI) and a eutrophication model (EUTRO). The eutrophication model was based on a simplification of the Potomac Estuary Model [31] (PEM). It allowed simulation of up to eight state variables (Figure 8) that could be considered as four interacting systems: phytoplankton kinetics, the phosphorus cycle, the nitrogen cycle and the dissolved oxygen balance. The general WASP mass balance equation was solved for each state variable. This kinetic construct was retained for WASP Versions 3–6.

The toxicant model in WASP3 was based on TOXIWASP (Figure 6 above). For WASP4, TOXIWASP and WASTOX were modified and refined and the toxicant routine rewritten resulting in TOXI. TOXI simulated the transport and transformation of one to three chemicals and one to three types of particulate material (solid classes). The three chemicals could be independent—or they may be linked with reaction yields, such as a parent compound–daughter product sequence. Each chemical existed as a neutral compound and up to four ionic species. The neutral and ionic species could exist in five phases: dissolved, sorbed to dissolved organic carbon (DOC), and sorbed to each of the up to three types of solids defined by the user (e.g., sand, silt, fine silt, clay). Local equilibrium was assumed so that the distribution of the chemical between each of the species and phases was defined by distribution or partition coefficients. In this fashion, the concentration of any species in any phase could be calculated from the total chemical concentration. Therefore, only a single state variable representing total concentration was required for each chemical. The TOXI model was then composed of up to six systems, three chemical and three solids, for which the general WASP mass balance equation is solved. This kinetic structure for the toxicant model remained relatively unchanged for WASP4 through WASP6.

#### 2.3.5. Auxiliary/Support Programs

These included the pre- and post-processors described above. In addition, beginning with WASP4, the DYNHYD hydrodynamic model was released with the WASP model (DYNHYD3 for WASP3 which was refined to DYNHYD5 released with WASP5). The DYNHYD model is a simple hydrodynamic model that simulates variable tidal cycles, wind and unsteady inflows. It produces an output file that can be linked with WASP to supply the flows and volumes to the water quality model. That is although released with WASP and described in user documentation it was a separate standalone model. The DYNHYD model is still provided as a component of stream routing modules in the present WASP model [32].

#### 2.3.6. Model Linkage

**Figure 9.**
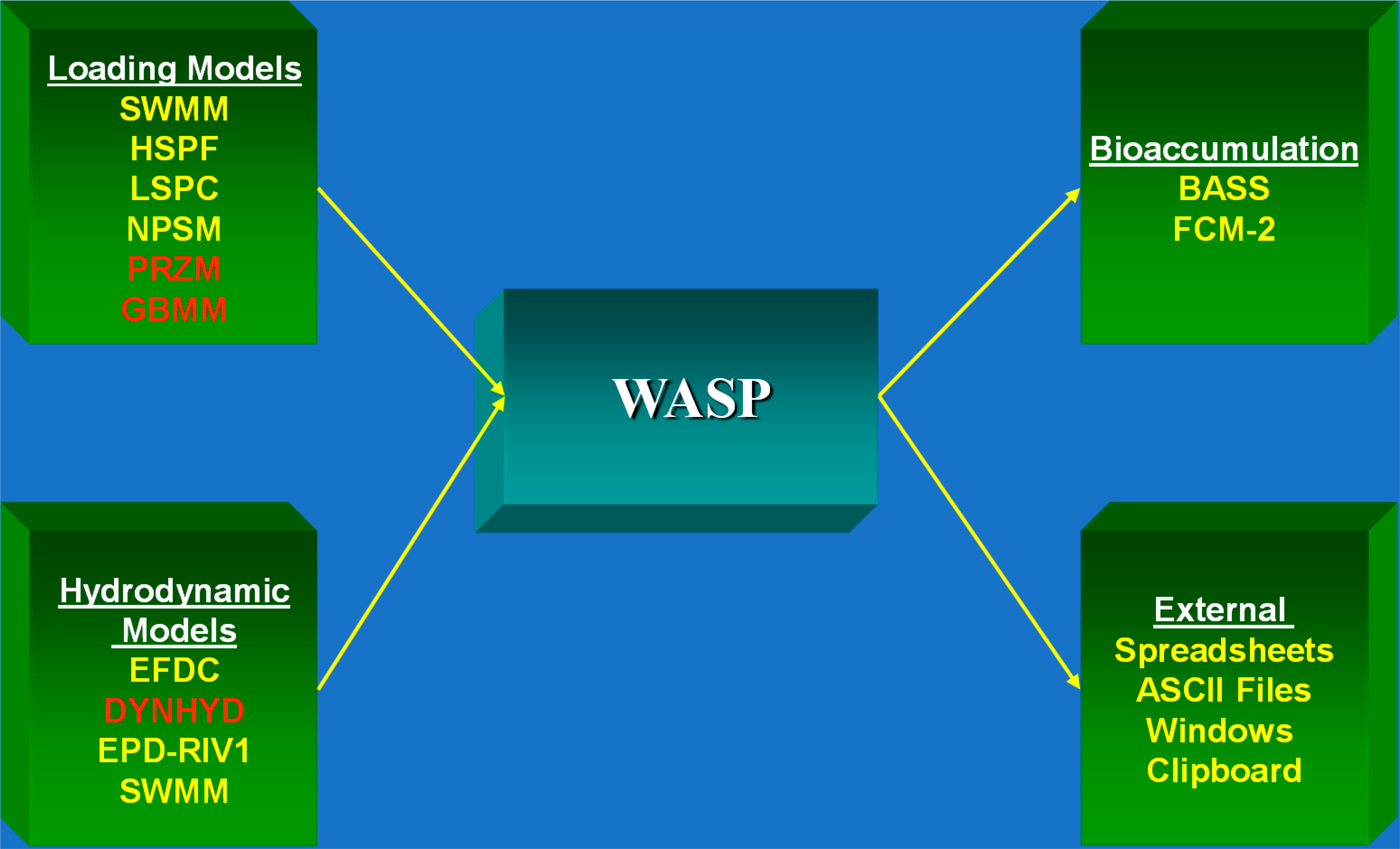
Example of WASP linkages.

During this period WASP was commonly linked with hydrodynamic models such as DYNHYD. The linkage consisted of the hydrodynamic model writing an output file containing transport information (e.g., volumes, flows, velocities, etc.) which were then read and used by WASP. A variety of other linkages occurred during this period. In addition, given the nature of the issues associated with TMDLs and toxicants, linkages with hydrologic (loading) models, fish bioaccumulation model (BASS, FCM, etc.) and others were common (Figure 9).

### 2.4. WASP6 (2001) and WASP7 (2012)

#### 2.4.1. Water Quality/Regulatory Environment

**Figure 10.**
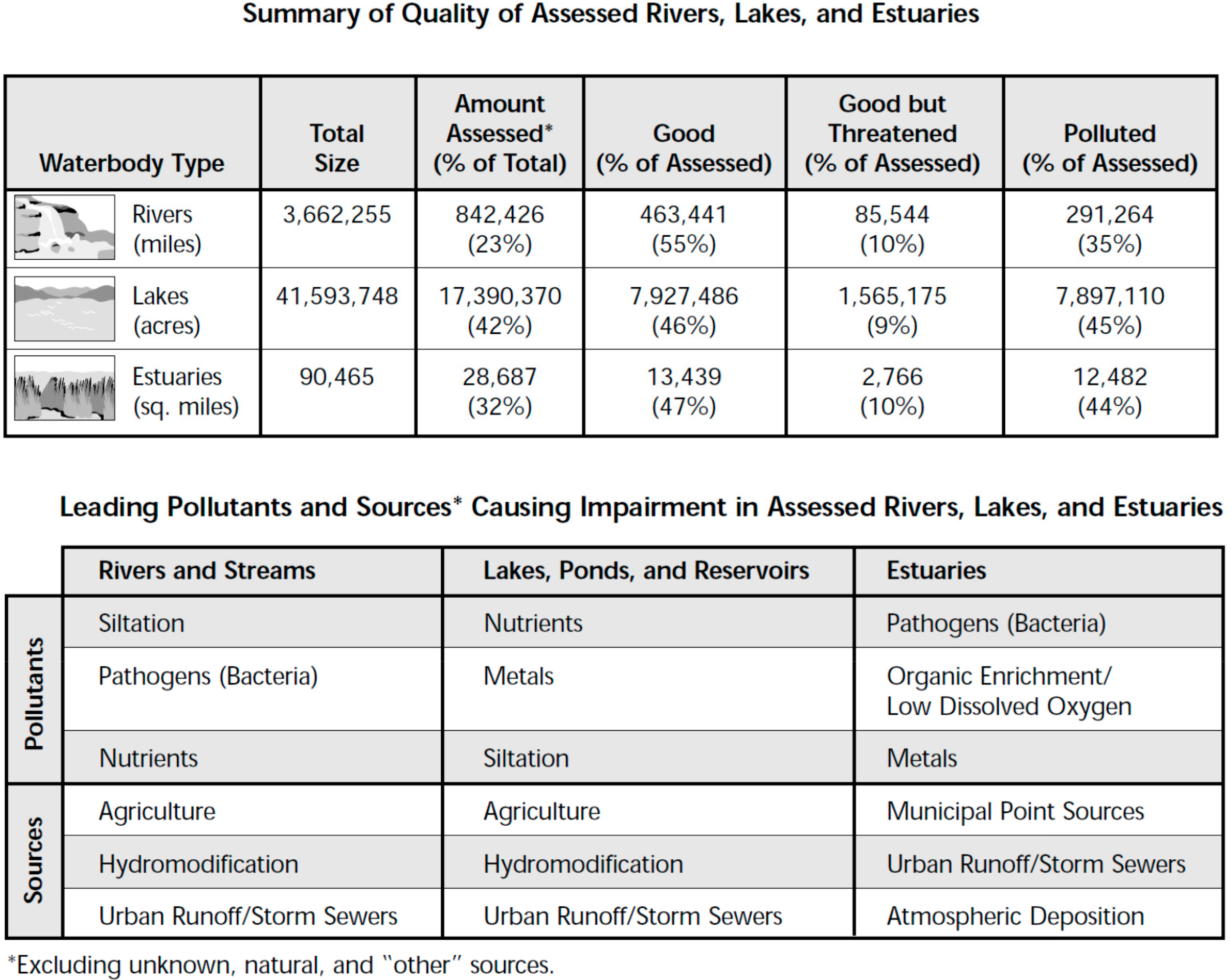
Water quality conditions in the United States, a profile from the 1998 National Water Quality Inventory Report (303(d) list) to Congress [35].

TMDLs continued to be a major regulatory issue in the 2000s and both conventional and toxic chemicals were of concern as illustrated from the results of the 1998 303(d) list of impaired waters (Figure 10). As part of the TMDL process, states were required to identify waterbodies not meeting water quality criteria, report those to the USEPA and Congress, establish a priority ranking and the perform TMDLs for those waterbodies and chemicals of concern. During this decade, given the large number of impairments, TMDLs became a major focus for many regulatory agencies.

During this period nutrients, also a major cause of impairments, became a major issue. At that time, and still today, states had in general not developed numeric criteria for nutrients so that impairments as listed in the 303(d) report (Figure 10) were largely based on narrative standards (“free from” standards; e.g., a criterion that describes the desired conditions of a water body being “free from” certain negative conditions). In 1998 the USEPA published a national nutrient criteria strategy. In January 2001, the USEPA published a series of criteria documents for nutrients in lakes and ponds and rivers and streams based on ecoregion. In addition, during this period, sediments for which most State’s did not have numeric criteria were identified as the leading cause on impairments in the 1998 303(d) list (of waters not meeting water quality criteria).

During this period, while organic pollutants remained an issue, metals (other than mercury) and mercury were identified as major environmental pollutants. In 1997 the USEPA released an eight-volume report to congress on mercury [33] and in 2000 the National Academy [34] produced a report on the toxicological effects of methylmercury which raised awareness in the U.S. of the impacts of mercury in the environment.

#### 2.4.2. Programming Environment

**Figure 11.**
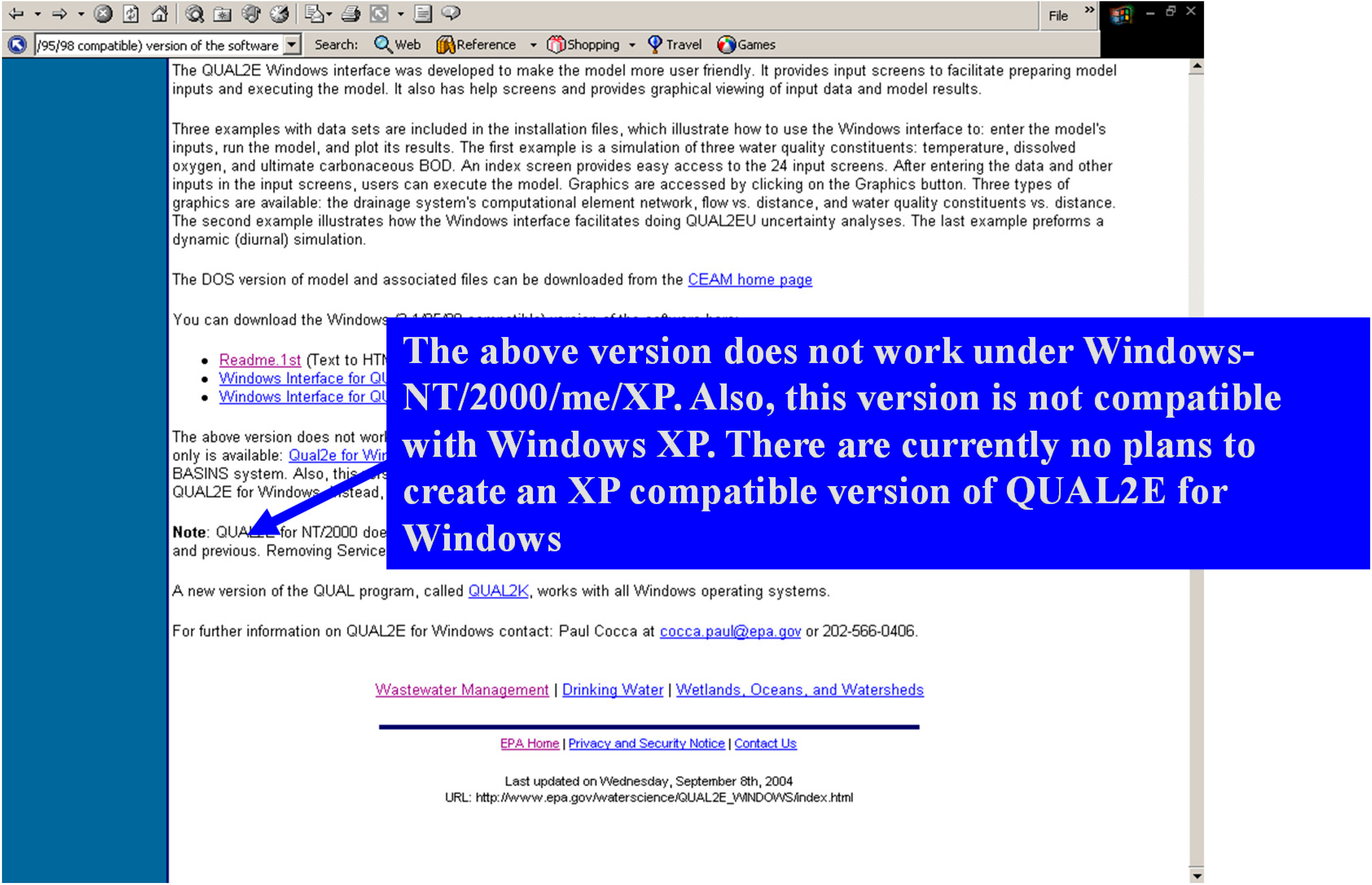
Screen capture of United States Environmental Protection Agency (USEPA) distribution website for QUAL2E from September 2004 with specific text extracted and highlighted.

During this period of development, Windows rapidly evolved from the original 1985 version to Windows 95 (1995) to Windows 98 (1998) to Windows 2000 (2000), followed by ME, Windows XP (2001) and Vista in 2006. Windows 7 was introduced in 2009, followed by Windows 8 in 2012. WASP was originally developed under IBM’s DOS (Disk Operating System) which later became Microsoft DOS (MS-DOS). Early versions of Microsoft Windows supported MS-DOS, while later versions supported an MS-DOS emulator. However, with versions Windows-NT/2000/me/XP, MS-DOS was no longer supported. That meant that WASP and other models needed to be converted from a MS-DOS to a Windows environment—a non-trivial translation that many other models were not able to make. In addition, each new version of Windows often required new modifications; various versions of Windows were not necessarily backwards-compatible, so adapting to new versions of Windows typically required some changes in the architecture of computer models. This resulted in a number of very useful and relevant models going away because either the developers or the agency supporting the model failed to support its translation between Windows environments. A classical case was the QUAL2E model [36], which was once the most widely used waste load allocation model in the world and was distributed by the USEPA. Originally based in MS-DOS (as was WASP), as Windows evolved from its original version to those (such as Windows 2000) which no longer supported MS-DOS, QUAL2E became unusable. Rather than updating the model, the USEPA elected to discontinue its use and distribution in 2004 (Figure 11) even though it would still have been useful in today’s regulatory environment. The void created by the absence of QUAL2E was filled by a new model, which is also described in this special issue, QUAL2K [37–39].

An additional restructuring of WASP 6 and 7 for input and output was based on the linkage between input and output and the Paradox database structure. In the modified WASP6 and WASP7 input, information on the model structure such as the state variables associated with each model type and their associated parameters, time functions and kinetic constants, could easily be stored in a database structure and retrieved depending on the model and variables selected by the user. Time varying input information (e.g., boundaries and loads) for specific WASP modules could be included in a database structure, based on the Paradox or spreadsheets, and accessed during runtime. Therefore, input time series no longer had to be specified in a time series of model input but could be obtained from an external Paradox database linked to WASP. Similarly, output information for comparison of observed data with model predictions could be in a Paradox database format. Unfortunately, Paradox was discontinued so that further development of WASP7 with database linkages was not possible.

#### 2.4.3. User Interface (Input/Output)

The period of development of WASP6 and WASP7 also included a Windows-based graphical user interface (GUI) for constructing input datasets and managing simulations as documented by Wool et al. [40]. Converting WASP from the MSDOS environment to MS Windows required structuring of the code and development of a new preprocessor and post processor. A mixed language approach was adopted for the restructuring of WASP under Windows. The graphical user interface and the post processor were developed using C++ language. The scientific modules stayed in their native FORTRAN environment. This approach allowed the interface to take advantage of a more structured and robust language for dealing with large data such as C++, while allowing the scientific modules to remain in FORTRAN which is more robust at numeric calculations.

**Figure 12.**
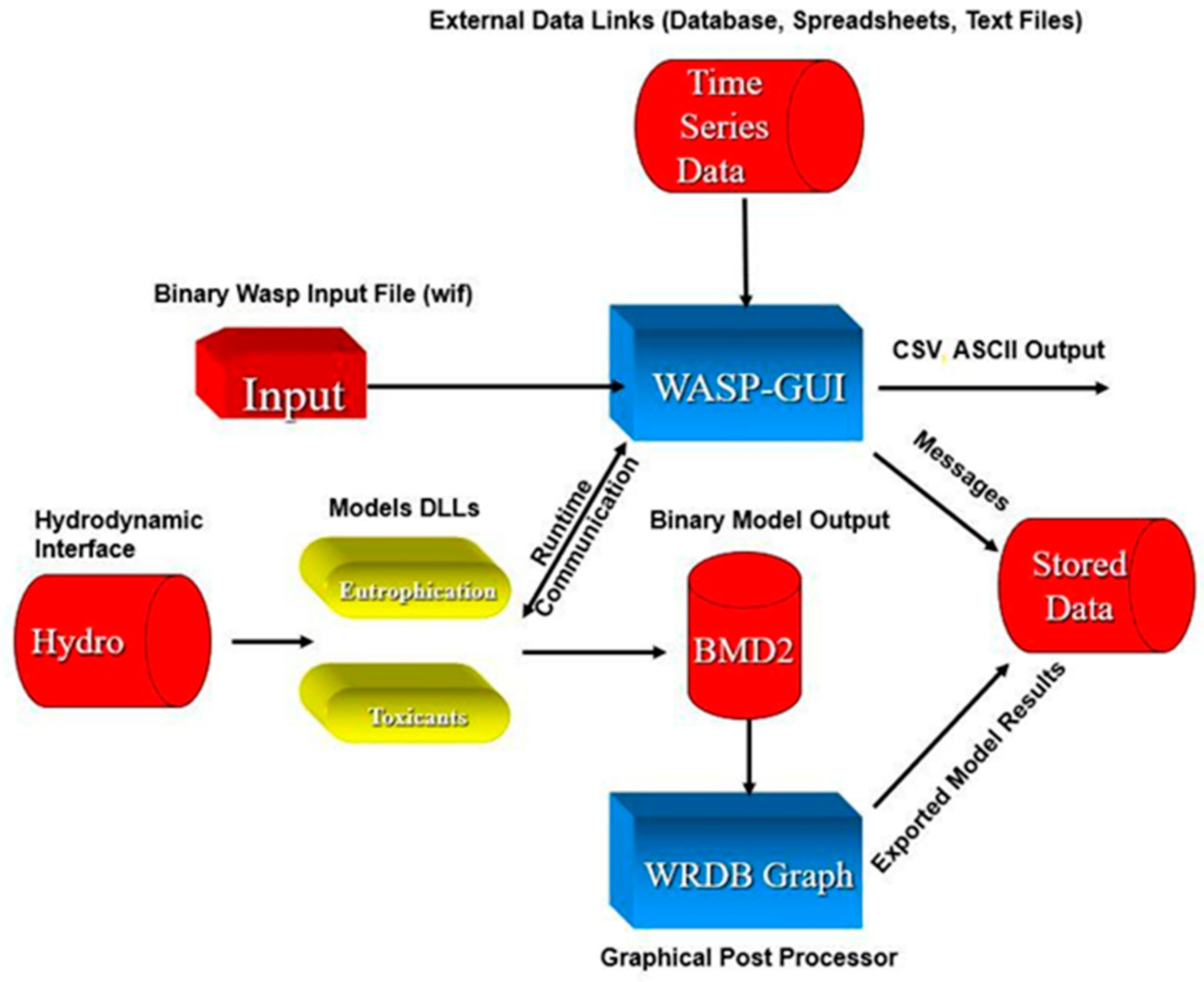
WASP6/7 model structure.

Figure 12 below illustrates the models restructuring and how the individual components relate. Redeveloping WASP under the Microsoft Windows environment afforded the opportunity to use some more advanced programming tools. One of the biggest advances and improvements to the WASP user interface was the introduction of controlling content and capabilities of the WASP modeling environment via a database file. This database file would contain all the information that is displayed to the users’ specific to the model they were running.

The decision was made to turn all the scientific modules (i.e., the eutrophication and toxicant model) into dynamic link libraries. They would not be a standalone executable, but a dynamic link library that could be called directly from the user interface. This approach allowed the rapid development of the graphical user interface because it no longer had to rely on creating an ASCII input file that could be read by the previous wasp executables. A unique approach was developed to transmit input information to the DLL models at runtime using inter-process communication. Once the user hits the execute icon and the appropriate model DLL is loaded, it begins to query the user interface for the information that is needed. Furthermore, the graphical user interface would serve as a time series server to the model while it was simulating through time. This meant that the graphical user interface would be interpolating each individual time series to determine what value should be passed to the model at the given simulation time. This approach allows WASP to have an unlimited number of time series and data points that could be used in any given simulation.

The development team developed several other dynamic link libraries that would help facilitate linking WASP to other tools and storing model results in a format that could be rapidly rendered in a graphical post processor. The end result of the restructuring of the WASP environment is that the modeling tool itself no longer reads or writes files, resulting in a drastic improvement in runtime and the ability to review model results quickly.

Previous versions (through WASP5) still required that the user develop an ASCII text input file-based card images for WASP. This file was formatted so a number in the wrong place could result in substantial errors. The burden of creating the input led users to spend a large portion of the application time on creating and editing this input file rather than running the model. The goal of the restructure was not to rely on the user to know where individual data should be placed in a file. The Windows graphical user interface would maintain an internal data structure of all information provided by the user and serve it to the specific WASP module (eutrophication/toxicants) when run. Adopting this approach allowed future versions of WASP to be backwards compatible with previous releases due to versioning in the WASP binary input file. The creation of the Windows-based interface greatly reduced the effort made in creating input files. Time varying input could also be linked with external files, and the required information read from those files at runtime.

A graphical post-processor (MOVEM) was also developed for and distributed with WASP. MOVEM provided the capability of generating multiple x/y line plots of predicted and/or observed model results, where the observed data were obtained from a compatible database. MOVEM could also store plot configurations making it easy for users to create graphs for comparison with observed data or previous model runs such as during model calibration or for reporting. One of the difficulties in having a complex grid (e.g., Figure 15) is that it is difficult to interpret results using x/y line plots alone. MOVEM also allowed the modeler to display the results in a two-dimensional rendition of the model network where the model network was color shaded based upon the predicted concentrations. MOVEM also provided for animation of model results, aiding in the interpretation of both spatially and time varying model predictions. MOVEM and the preprocessors, represented a major advance in the evolution of WASP. A graphical user interface User’s Guide [40] was distributed with WASP.

#### 2.4.4. Kinetic Structure

The evolution of previous versions of WASP was driven primarily by changes in computer architecture, more specifically Windows and the IBM PC, the regulatory environment (e.g., TMDLs) and the parallel development of hydrodynamic and hydrologic models. In previous versions the water quality kinetics for toxicants and eutrophication remained relatively unchanged. As issues and capabilities changed, however, WASP6 and WASP7 incorporated new water quality algorithms as described in WASP user documentation listed below.

- Benthic algal model [41]
- Temperature and coliform bacterial model [42]
- pH Alkalinity model [43]
- Multi-algal model [44]
- Sediment diagenesis model [45]
- Metal speciation (META4) [46] (not presently incorporated into WASP7 or WASP8)
- Mercury model [47,48]

The primary WASP models TOXI and EUTRO were retained with additional capabilities. The toxicant code, TOXI, was combined with separate databases to support four modules–simple toxicant, non-ionizing toxicants, organic toxicants and mercury. The simple, non-ionizing and organic toxicant models were based on the original toxicant model in WASP from WASP4, but model database developed so that the user only saw some subset of the input depending on their selection. The WASP mercury module simulates elemental mercury, Hg^0^, inorganic divalent mercury, Hg(II) and monomethyl mercury, MeHg. Mercury species are subject to several transformation reactions, including oxidation of Hg^0^ in the water column, reduction and methylation of Hg(II) in the water column and sediment layers and demethylation of MeHg in the water column and sediment layers. If the user selected the mercury model, they saw in the graphical user input only those input relevant to mercury, although the mercury model was largely based on the original TOXI model.

**Figure 13.**
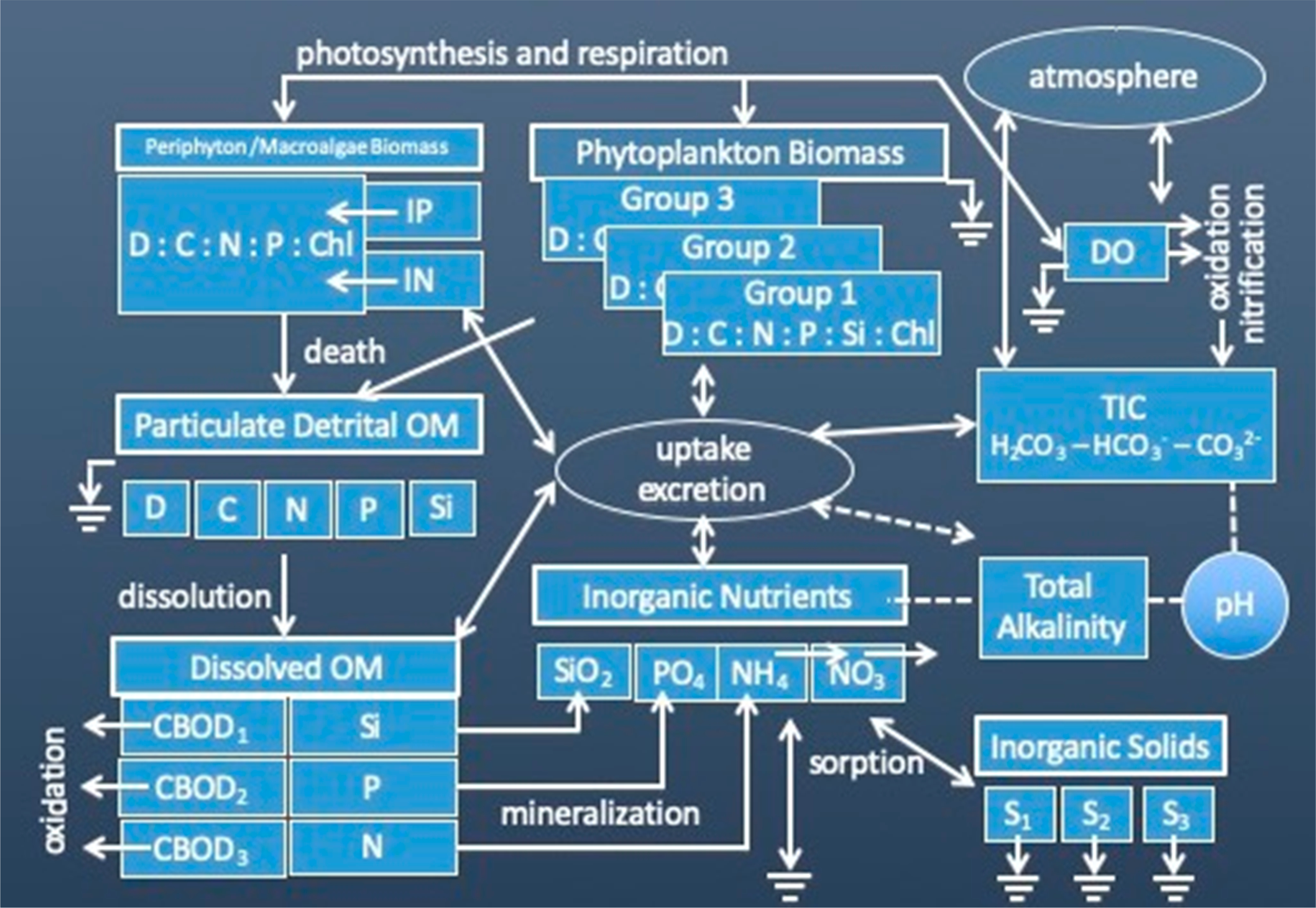
Advanced eutrophication kinetic model for WASP7.

The EUTRO model included additional state variables and the user could elect to use either a simplified or advanced eutrophication model. While the simple eutrophication model had the basic construct of the original EUTRO of WASP Versions 3 through 5, the advanced model included the additional capabilities listed above–three algal groups, macroalgae and benthic algae, pH and alkalinity and sediment diagenesis. Organic matter was broken out into particulate and dissolved state variables for total dry weight, carbon, nitrogen, phosphorus and silicon. The resulting kinetic structure is illustrated in Figure 13.

**Figure 14.**
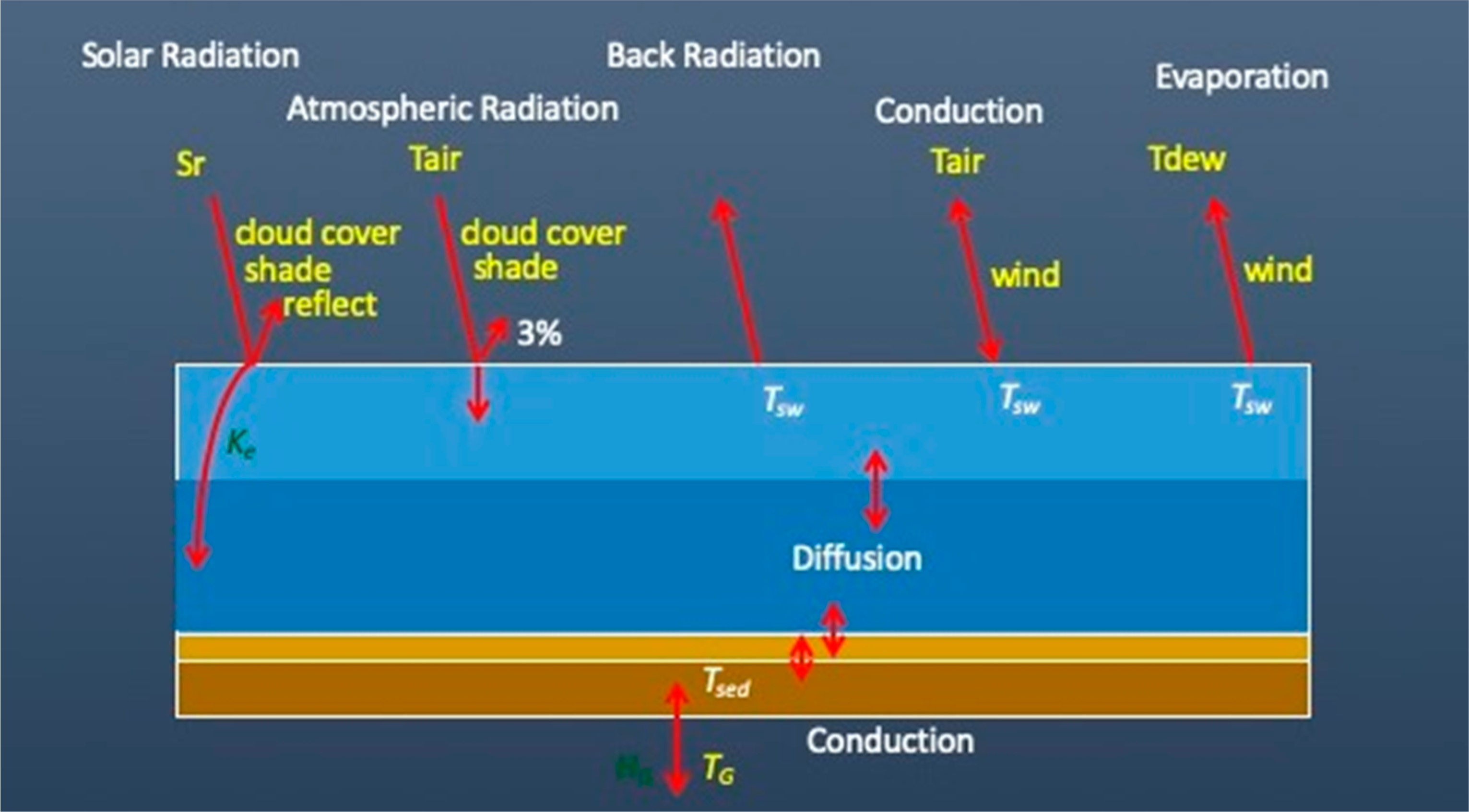
Heat exchange processes. Sr is solar radiation, K_e_ is light extinction coefficient, Tair is air temperature, Tdew is dew point temperature, T_sw_ is water surface temperature, T_sed_ is sediment temperature and T_G_ is ground temperature.

In order to simulate water temperature, a separate heat balance module, HEAT, was developed and incorporated into the WASP structure. Water temperature is simulated accounting for a set of energy balance processes as shown in Figure 14. Specified forcing functions are shown in yellow. Solar radiation is attenuated above the water surface by cloud cover, vegetative and topographic shading and reflection. The remaining radiation is attenuated down through the water column, adding heat. Atmospheric radiation is also attenuated by cloud cover, shade and reflection, and adds heat to the water surface. Back radiation from the water surface releases heat back to the atmosphere. Heat may be conducted between the atmosphere and water surface. Evaporation releases heat from the water surface back to the atmosphere. Finally, ground temperature adds or removes heat to the bottom sediment layer by conduction.

An additional feature incorporated into WASP7 at this time was the ability to pass information from one kinetic model to another. While water temperatures could still be specified in EUTRO, now they could be simulated in HEAT and passed to EUTRO. Further, those temperatures along with pH, algal production, and other variables simulated in EUTRO could be passed along to TOXI for use as forcing functions in contaminant simulations. These additional features made WASP much more adaptable to problems ranging from mercury contamination to acidification to harmful algal blooms and many others.

#### 2.4.5. Auxiliary/Support Programs

A variety of support programs were also developed for and distributed with WASP at this time for purposes such as making multiple runs for WASP.

#### 2.4.6. Model Linkage

**Figure 15.**
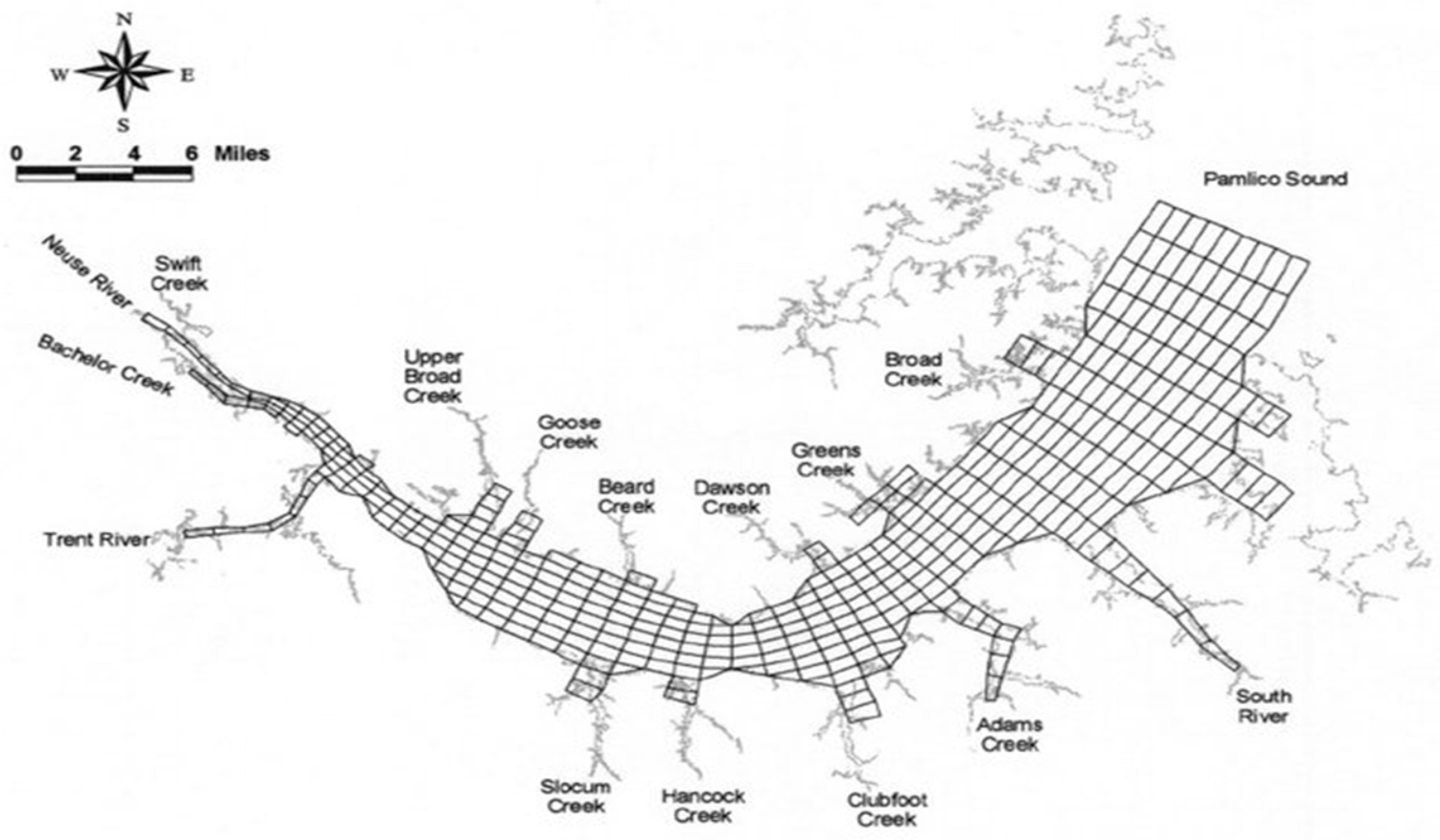
Hydrodynamic and water quality model segmentation for the Neuse River Estuary [4].

Linkages with other models, obtaining information from or providing information to, became the norm during this period, largely driven by the need for including non-point sources for TMDLs. In addition, the parallel development of hydrodynamic models resulted in the norm being predicting (using these external models) rather than specifying transport. Some hydraulic models were incorporated directly into the WASP framework and distributed with WASP, such as the DYNHYD model and a kinematic wave model [32]. During this period processing speed made the application of complicated three-dimensional hydrodynamic models feasible. The evolution of the EFDC model [49,50], also described in this special issue, led to commonly using EFDC and WASP in tandem for many water quality studies, in particular for estuaries with complicated hydrodynamics. One example is the application by Wool et al. [4] to the Neuse River Estuary in North Carolina. That application involved hydrologic models (The Hydrologic Simulation Program–FORTRAN, HSPF, Nonpoint Source Model, NPSM, in conjunction with U.S. EPA Region 4’s Watershed Characterization System) along with EFDC and WASP. The numerical grid for the EFDC and WASP application is illustrated in Figure 15 and consisted of 405 horizontal grid cells with four layers for a total of 1620 cells (e.g., WASP segments), which is a big change from the few cells and descriptive transport used in the early applications of WASP to the Great Lakes (e.g., Lake Ontario, Figure 4).

#### 2.4.7. Solution Technique

The accurate numerical solution of the three-dimensional transport equations requires advanced solution techniques. When linked to EFDC, WASP implements the EFDC numerical scheme, COSMIC, a highly efficient finite difference semi-implicit solution scheme, which is second order accurate in space and time [50]. COSMIC uses a mass conservation fractional step solution scheme for the Eulerian transport equations at the same time step or twice the time step of the momentum equation solution [51]. The advective step of the transport solution is based on a flux corrected transport version of Smolarkiewicz’s multidimensional positive definite advection algorithm, which is monotone and minimizes numerical diffusion. The horizontal diffusion step, if required, is explicit in time, while the vertical diffusion step is implicit.

#### 2.4.8. Hydrodynamic Linkage API

**Figure 16.**
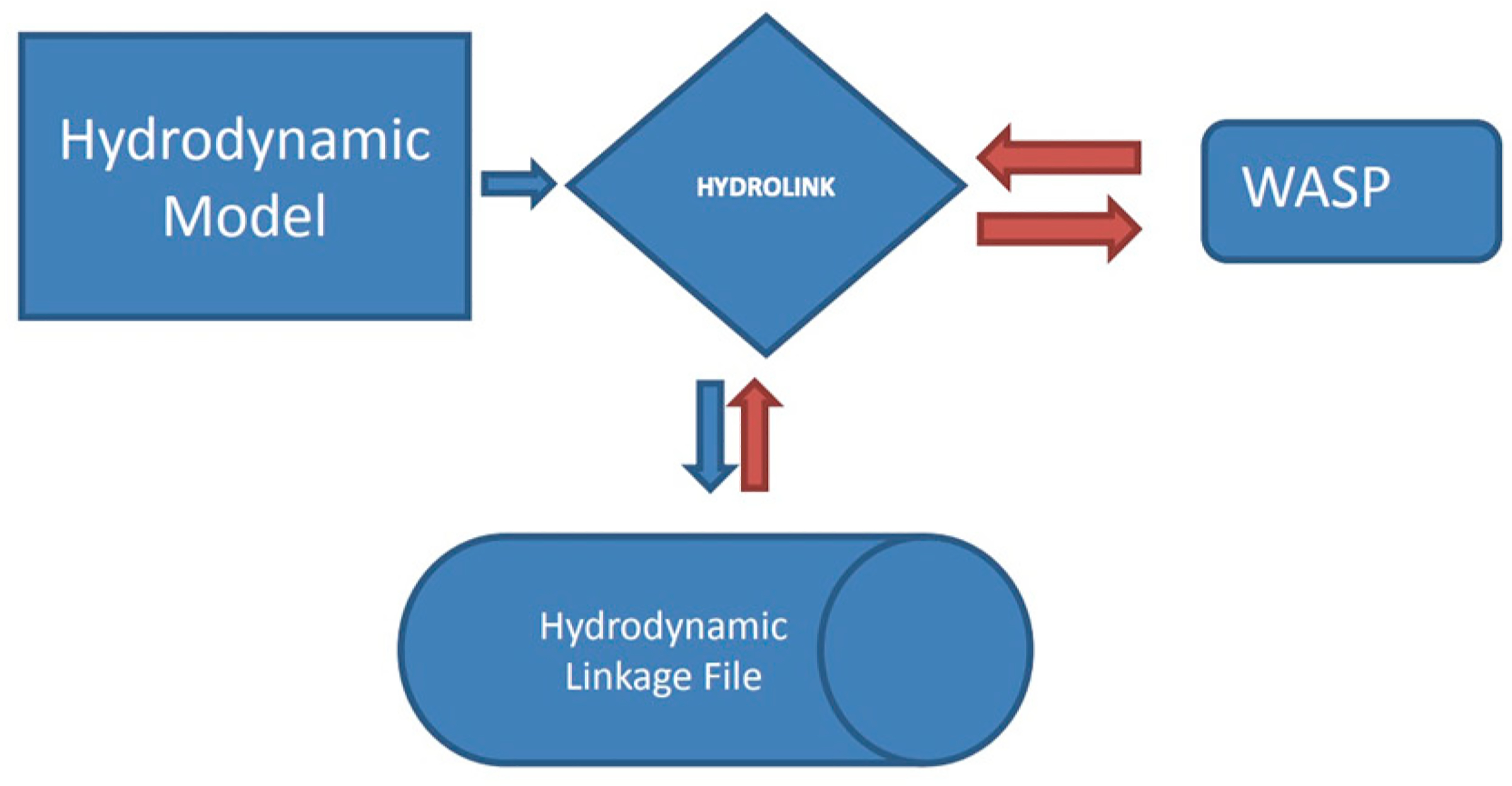
Hydrodynamic linkage application program interface.

With the increase in computing capabilities, WASP users were looking to apply the model where detailed spatial and temporal resolution was important for managing water quality. The ability to apply two- and three-dimensional hydrodynamic models to large water bodies became commonplace. While WASP had the ability to receive hydrodynamic information from DYNHYD, the linkage was specific to DYNHYD’s output. A generic method for linking 1/2/3 dimensional hydrodynamic/hydraulic models was developed. This involved the development of an application program interface (API) to facilitate the generation of a hydrodynamic linkage file that could be used by WASP. The API was developed in such a manner that it could be used by virtually any transport model, including spreadsheet calculations. The Figure 16 provides an overview of the API. The core of the API is a shared dynamic linked library (HYDROLINK.DLL).

This DLL is utilized by both the hydrodynamic model and WASP, which ensures that information is passed correctly from the hydrodynamic model to WASP. Currently the API assumes that the hydrodynamic and WASP models are linked 1::1 in time and space. There are no algorithms within the API to collapse a grid in space or time. This means whatever spatial component used in the hydrodynamic model (cell, node, segment) is represented by a segment in WASP. The spatial and temporal information that is passed by the hydrodynamic model to WASP includes segment volumes, depths, velocities, temperature and salinity. The hydrodynamic model also passes the individual flows that are calculated across all cell interfaces. All information passed from the hydrodynamic model and eventually used by WASP is done via pointers in memory. Because neither the hydrodynamic model nor WASP is reading or writing files, the creation/use of the linkage file does not affect model performance. Furthermore, the amount of information that can be potentially passed to WASP can become quite large because the API compresses and decompresses that data at runtime. On average this reduces the size of the linkage file by 80%.

The models that have been successfully linked to WASP include: EFDC (1/2/3-dimensions), DYNHYD (2-D lateral), HEC-RAS (1/2-dimensions), ROMS and CH3D (3-dimensions).

### 2.5. WASP Today (WASP8) (2012–Present)

The development of WASP8 allowed the integration of all the successes and failures of the past to be re-implemented in a new modern computing environment. When WASP6/7 was developed, proprietary development tools were used to program the Windows graphical user interface, the graphical post-processor and to some extent the FORTRAN scientific modules. Support for Linux and Mac did not exist in WASP 6/7. The proprietary development tools used for WASP6/7 are no longer available and are not supported in the current Windows environment. This required a major redevelopment. The WASP program structure of version 6/7 remained unchanged for 16 years. The release of WASP8 marked the largest restructure and reorganization of the modeling system since WASP6. The major changes in WASP8 are presented below. The WASP model is distributed free of charge by the USEPA Center for Exposure Assessment Modeling (CEAM) [52].

#### 2.5.1. Water Quality/Regulatory Environment

WASP remains as one of the most widely used water quality models in the United States and throughout the world. It is continually enhanced to meet the demands of the regulatory environment. WASP was the water quality model used by EPA to determine numeric nutrient criteria for estuarine waters in the State of Florida. WASP was linked with EFDC (hydrodynamic model) and LSPC (watershed model) to determine appropriate nitrogen and phosphorus concentrations to protect the designated uses of 13 estuaries in Florida.

The enhancement of the organic chemical model to include nanochemicals and solids allows regulators to investigate the impact of emerging contaminates such as pharmaceuticals, micro plastics and more complex organic chemicals and metals.

#### 2.5.2. Programming Environment

Because the development tools that were used for WASP6/7 were no longer available or supported by Microsoft Windows, we decided to use all open source publicly available development tools and libraries for the development of WASP8. The graphical user interface is still written in C++ and can be compiled with open source toolchains. Because this approach was going to cause virtually a complete rewrite of WASP user interface and the supporting shared libraries, we decided to include the Mac OS X and Linux–Ubuntu environments along the traditional Microsoft Windows environment. We also decided to only support 64-bit machines. This allowed WASP to take advantage of the 64-bit architecture that was available to all the current computers.

The 64-bit machines give WASP access to more core memory, which allows it to consider more state variables and model segments, a major limitation under the previous versions of WASP developed for 32-bit Windows. WASP6/7 were developed and implemented using FORTRAN77 standards. The decision to go to the 64-bit environment gave good reason to update the FORTRAN code in the scientific modules to the most current standard. The scientific code was restructured to take advantage of dynamic memory allocation and to only allocate memory based upon the state variables the user selected to simulate. This is one of the largest advantages of WASP over most water quality models that are available today–the user controls which state variable to use and parameterize. For example, the user can construct an application using a subset of the 26 state variables available in the eutrophication module, such as dissolved oxygen, ammonia, nitrate and carbonaceous BOD. The user is not required to populate information of all the state variables the model is capable of simulating. The same approach was implemented in the graphical user interface, as the user adds the state variables to be considered the interface makes available only input requirements that are needed for the selected state variables.

#### 2.5.3. System Architecture

One goal of the modern system software architecture for WASP was to reach the broadest audience of users by supporting multiple operating systems (Mac, Ubuntu Linux and Windows). Another important goal was to use modern tooling while avoiding tool vendor lock-in by choosing open-source library and software package dependencies.

Towards these ends, the Qt GUI toolkit was chosen to implement the cross-platform WASP GUI. Qt is a broadly popular, well documented and stable open source development platform with a large support community. Qt abstracts the underlying windowing system, allowing WASP code to be largely platform agnostic, thereby enabling a single WASP code base to be built for Windows, Mac and Linux. Qt widgets and graphing components are used to render model input data within the WASP GUI.

Gnu Fortran and Gnu G++ compilers are widely available on every popular platform and were chosen to build all the WASP C++ and Fortran code. Gnu Fortran and Gnu G++ compilers are available on Mac, Linux and Windows.

In addition to these fundamental development toolchain building blocks, additional open source packages are utilized for graph plotting, data visualizations and database access.

Because of the multiple contributors to the development of the user interface and scientific models, all WASP source code is stored in a Git repository (an open source distributed version control system) and is integrated with an automated continuous-integration/continuous-delivery (CI/CD) system. The CI/CD system automatically builds and tests every new WASP source code revision, which helps to maintain high quality WASP software, as well as providing full traceability of all WASP release artifacts. The authors of WASP do not post the WASP source code to the general public, this is done to maintain the integrity of the model. All underlying equations used in the model are documented in users’ manuals or workshop materials. The WASP developers have always worked with the user community to make enhancements to the modeling framework.

#### 2.5.4. Major Components of the WASP Software Suite

**Figure 17.**
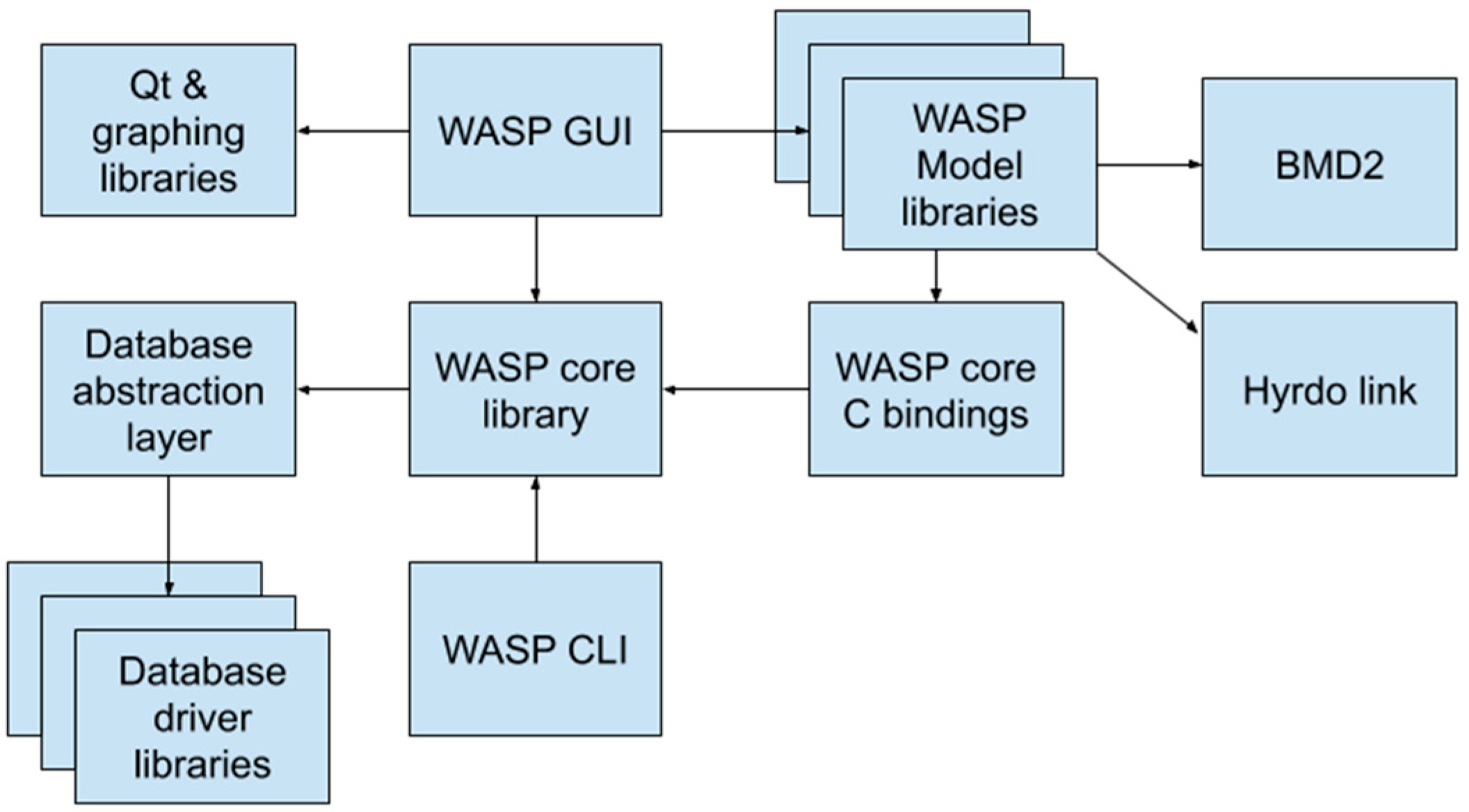
WASP suite components dependency diagram.

WASP is composed of a set of modular components (Figure 17). The main WASP UI is a Qt GUI executable. A command-line client is also provided. Each scientific model is a separate dynamically loadable library. Lower level dynamic libraries provide read/write interfaces for WASP input and output data formats.

### 2.6. WASP GUI

The WASP graphical user interface (GUI) is a Qt-based application with which the user interacts to prepare the WASP input data. The user sets up the WASP model input parameters by entering data into tables and dialogs within the GUI. The WASP GUI interacts with the WASP core library to query databases, read and write WASP input data, and to handle the execution of the WASP model.

### 2.7. WASP CLI

The WASP Command Line Interface (CLI) provides an alternative interface for using WASP in a command shell environment or scripting language. In a similar manner as the WASP GUI, the WASP CLI interacts with the WASP core library to query databases, read WASP input data, and to handle the execution of the WASP model.

### 2.8. WASP Core Library

The WASP core library is used by the WASP GUI and WASP CLI to handle the reading and writing of the WASP input data, as well as database queries and other core functions such as loading and executing WASP models. The WASP core library stores WASP input data in the binary WIF format. This binary format not only allows for more efficient data processing, but also enables the storage of significantly larger amounts of data more efficiently in the same space than a similar ASCII format dataset.

#### 2.8.1. WASP Model Libraries

The WASP model libraries are dynamically loadable libraries containing the scientific WASP model code. The WASP model libraries are loaded and executed by the WASP core library. The WASP model libraries call the WASP core C bindings to access the WASP input data. The pluggable modular architecture of the WASP model libraries provides the facility to easily support future scientific models.

#### 2.8.2. WASP Core C Bindings

The WASP core C bindings provide a C-compatible Application Binary Interface (ABI) which allows for linkage from many popular languages such as C, Fortran & others. This interface is used by the WASP Model libraries to access the WASP core library functions.

#### 2.8.3. BMD2

The BMD2 component handles the reading and writing of BMD2 files, which store the model output results. The BMD2 component provides an API for other components, enabling them to efficiently store and load large volumes of data while optimizing the data layout format for subsequent analysis and processing.

#### 2.8.4. Database Abstraction Layer

The Database abstraction layer provides the WASP core library a uniform application programming interface (API) for a variety of underlying database driver client libraries. By providing this abstract interface, the database abstraction layer can support many common databases via a single interface, and support for new data sources may be more easily added in the future. Open source database client libraries are used to access the underlying databases.

#### 2.8.5. User Interface (Input/Output)

The graphical user interface was completely rewritten from version 7. Many of the features and functionality that were incorporated in the previous version are still in WASP8. Most of the text that is displayed in the graphical user interface is retrieved from a database and is based upon model type (Eutrophication or Toxicants) and model state variables that the user selected for their simulation. This approach allows the graphical user interface to only display the variables (kinetic constants, segment parameters and environmental time functions) that potentially need to be defined. While the WASP7 interface presents all the available state variables in the model, WASP8 has the user select the state variables that they want to consider.

WASP6/7 had the ability to link to external databases and files to read in time series information for flows, model boundary conditions, environmental time functions and pollutant loads. The methods for linking in WASP6/7 were very cumbersome causing the user to do a lot of repetitive functions to bring the data into the interface. The methods for linking to external data sources were completely rewritten to allow the user to rapidly link external databases, spreadsheets and text files to a WASP input file. There are two mapping functions that are built in that allow the user to define which parameters from a database file would be paired with a WASP model state variable and which station or data location would be matched up with a WASP segment. Once these mappings are complete the user interface will automatically populate all the time series data for the boundary conditions for all state variables from the external data source. With this mapping information the user interface will set up queries for every unique condition. The User has the option of querying the data into the WASP input file data structure or leaving it as a live query which is queried when the user executes the model. The addition of the live query option allows WASP to be applied in “real time” as data are being collected and processed.

#### 2.8.6. Batch Model

WASP8 has a command line batch mode that allows the model(s) to be run without the aid of the graphical user interface. In the previous version of WASP, the graphical user interface had to be loaded to facilitate the data serving to the model. This redesign allows the model to be run and data served without the aid of the graphical user interface. The user can run as many command line simulations simultaneously as they would like. The command line execution is integral to using the sensitivity/uncertainty interface that is described below.

#### 2.8.7. Sensitivity/Uncertainty Interface

**Figure 18.**
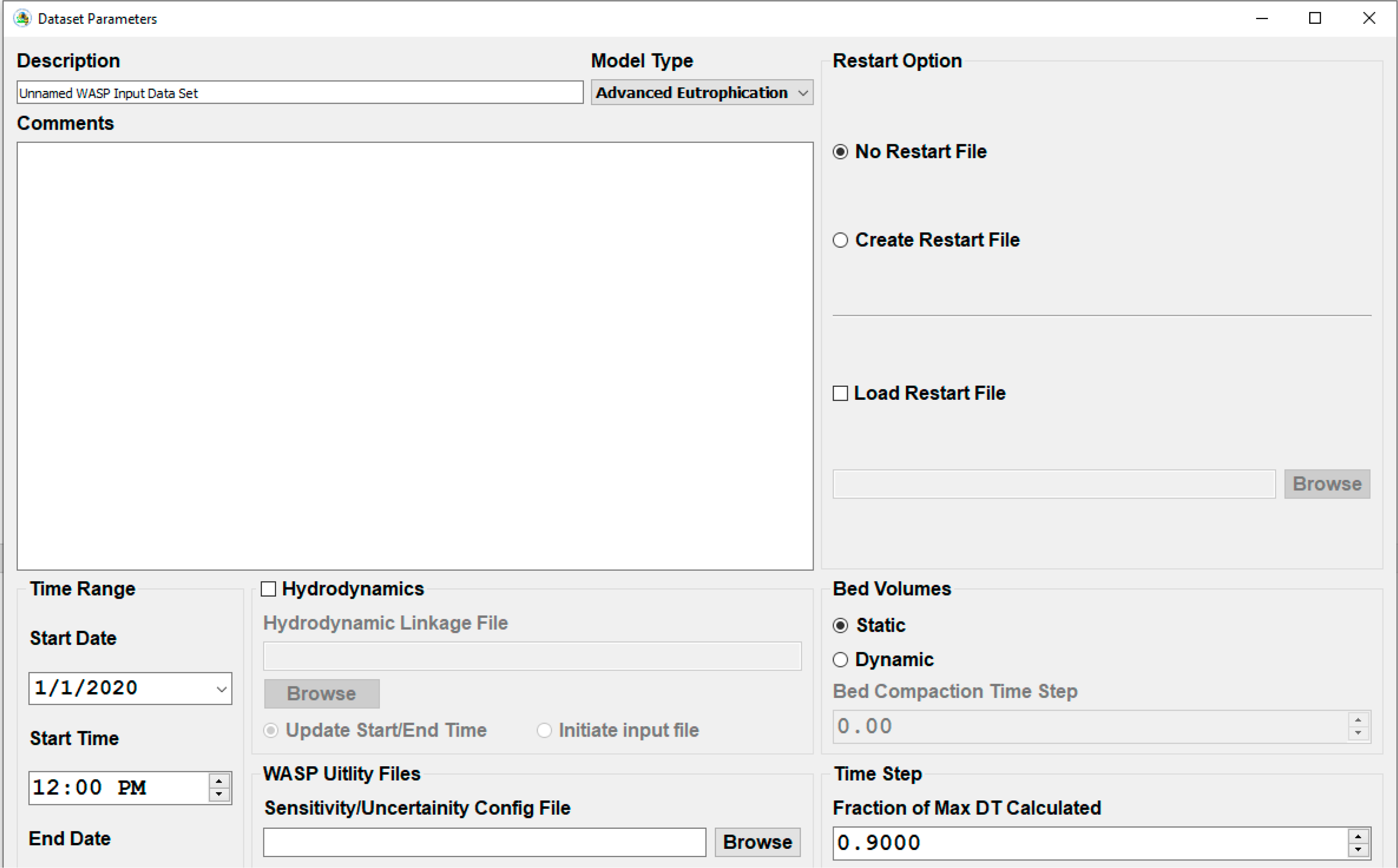
Sensitivity/ uncertainty configuration interface.

WASP includes an external interface for conducting advanced sensitivity analysis and auto calibration (Figure 18). This methodology utilizes a flat ASCII file that is ready at runtime. The user can change kinetic constants, segment environmental parameters and scale up or down advective flow and/or dispersive exchange scale factors. This functionality works both in running the graphical user interface or running from batch mode.

**Figure 19.**
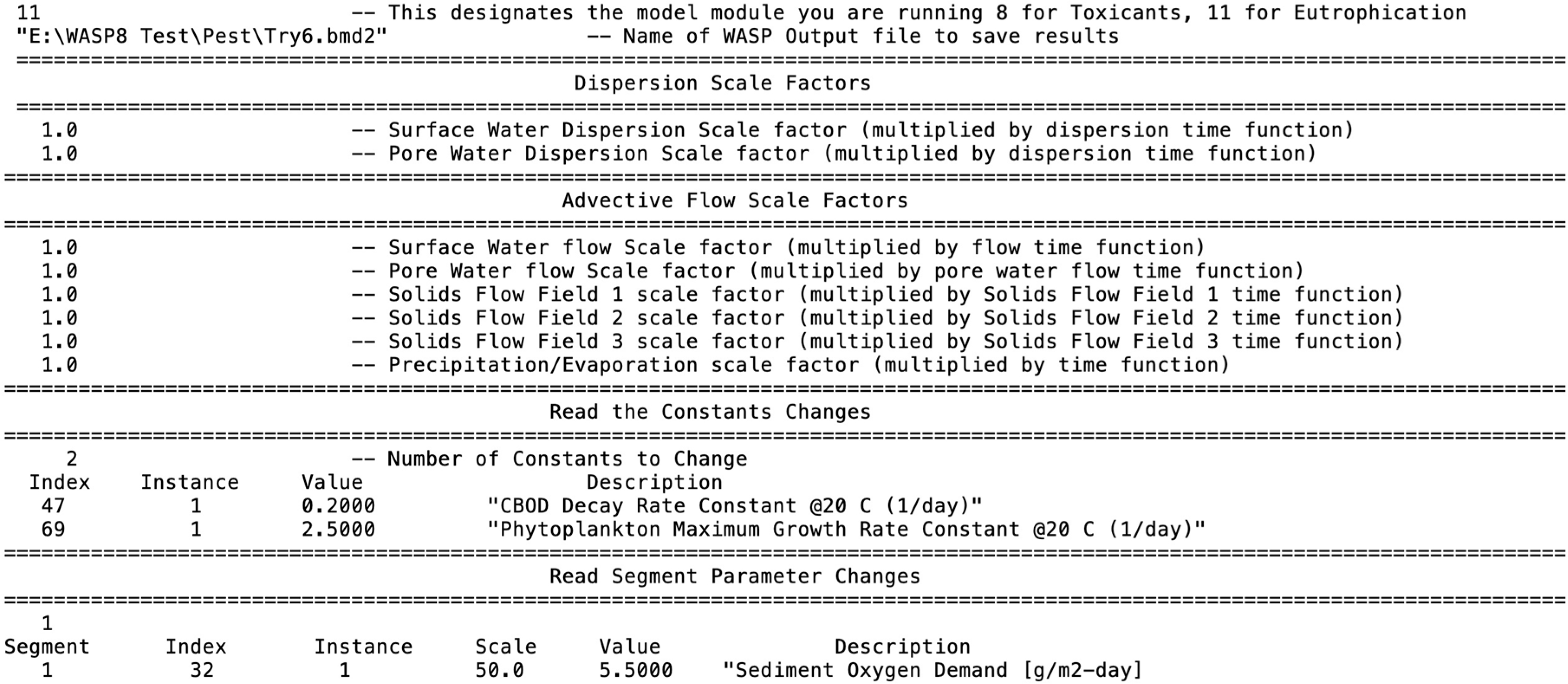
Example of the sensitivity/uncertainty configuration file.

This flat ASCII interface file (Figure 19) can automatically be altered from tools like the parameter estimation tool (PEST, [53]). Using this methodology, it is possible to make thousands of simulations varying these various components to aid in calibration or estimating uncertainty in some of the model assumptions.

### 2.9. Output

WASP6/7 utilized a binary output file (BMD) that was facilitated by a set of shared libraries. This output format worked well for the earlier versions of WASP. Several plotting packages were available to read the BMD file format and render plots and animations (MOVEM, WRDBGraph). With the increased capabilities of WASP8, more state variables and ability to simulate large model networks, it became apparent that the WASP6/7 BMD output format was not efficient enough to render plots quickly. For WASP8 a new version of output file structure was developed, again using shared libraries. This binary out file called BMD2 contains two copies of the model output, the first in the sequential structure as the information is stored as the model is simulating. Upon the completion of the simulation and before the model exits, a second copy of the data are processed and rearranged to allow rapid processing of the output. A plot of more than a million time series points can be rendered in less than a second. This optimized output structure meets the demands of simulating large networks over long simulation periods (10+ years). The BMD2 file format can still be processed using WRDBGraph and WRDBGis to plot time series data as well as spatial animations. WASP8 includes command line utilities that can be used to extract data from the BMD2 file into comma separated value files that can be read by Excel and other programs.

#### 2.9.1. Real Time Plotting/Animation

**Figure 20.**
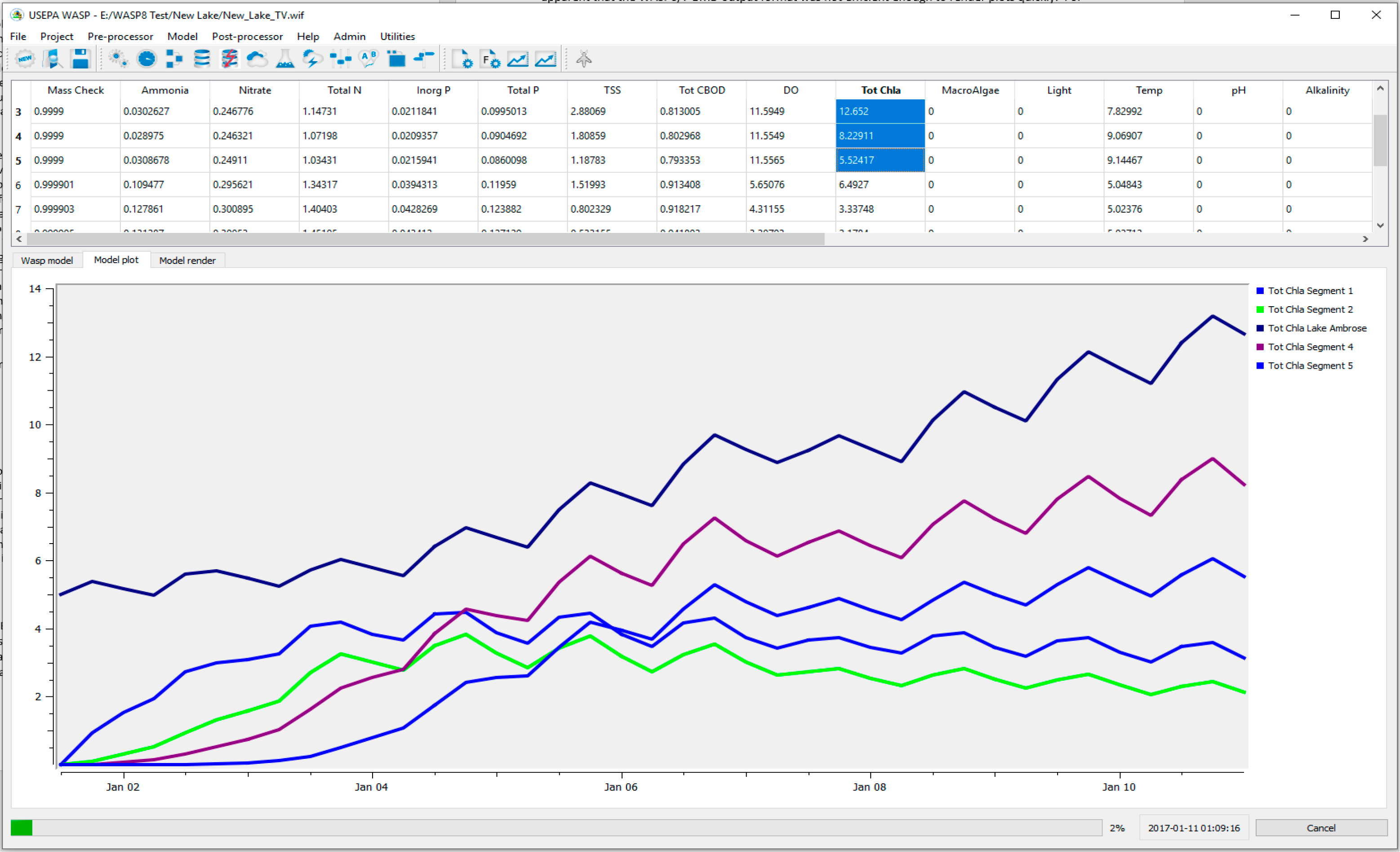
WASP real time plotting.

The most current release of WASP8 (Version 8.4) allows the user to interactively select model network segments and water quality constituents to plot during runtime (Figure 20). The functionality allows the user to inspect model results while the model is running its simulation. No longer does the user have to wait until the model run is completed to inspect model results. This feature allows the user to abort the simulation if they see on inspection an unrealistic or unexpected result.

The graphical user interface also has a model animation tab. This tab allows the user to load a user supplied shapefile that represents their model network. The interface uses color shading to illustrate the predicted segment concentrations for a selected water quality constituent. The spatial animation provides a good method to inspect large model networks to confirm transport integrity and expected model results.

#### 2.9.2. GIS Network Tool

With the release of WASP Version 8.4, a QGIS plugin is available for developing riverine model networks. QGIS was selected because it is an open source rapidly developing GIS platform that has user support throughout the world. QGIS is also available for all operating systems that WASP supports. This plugin replaces the WASP Builder Tool that is part of EPA’s BASINS GIS tool. This plugin takes advantage of the GIS capabilities of QGIS which will allow for a more efficient processing of the USGS NHDPlus national hydrography network. The plugin has the capability of downloading the NHDPlus coverage from the USGS data server for a user specified area and preprocess the coverage to develop a WASP model network. This tool allows the user to select the pour point of their WASP network and it will automatically trace upstream based on user specified parameters such as stream order. The plugin then smooths and recalculates all the average flows, velocities, depths and slopes to prepare the NHDPlus coverage for segmentation. The segmentation procedure considers travel time to determine how the riverine network is segmented to minimize numerical dispersion caused by irregular sized segments. Once the model network is developed in QGIS it is automatically pulled into the WASP user interface for the user to continue building their model.

The QGIS plugin can download data from any USGS stream flow gages located within the study area. The tool can use flow gage information outside the immediate study area using a drainage area ratio calculation to translate a regional gage to the study area. The tool will download the time series of flow for each gage selected, doing an appropriate units’ conversion and then process into a form that can be easily be used by the graphical user interface. While the QGIS plugin is being developed specifically to process data that is readily available in the United States, the methodologies developed in the plugin could be translated to similar datasets throughout the world. The plugin is written in Python 3 using PYQGIS QT framework. The source code for the plugin will be made available.

#### 2.9.3. Kinetic Structure

WASP8 features two main water quality kinetic modules—advanced eutrophication and advanced toxicant. The Heat module is no longer a separate module. In WASP8 it is an internal module linked directly within the eutrophication and toxicant modules so that water temperature can be selected and simulated along with the other selected water quality variables. As before, the user must specify several meteorological forcing functions, including solar radiation, cloud cover, wind speed, air temperature and wet-bulb temperature.

An internal light module was written to service the heat, eutrophication and toxicant modules in a consistent manner. Total solar radiation is either calculated internally (from latitude, date and time) or specified by the user as a time series. Total radiation is divided internally into three classes—ultraviolet (uVa and uVb, 3.6%), visible (46.4%), infrared (IR, 50%)—and eleven wavebands—uVb medium, uVb high, uVa low, uVa medium, uVa high, violet, blue, green, yellow–orange, red and near infrared. Default light extinction coefficients for water, chlorophyll, DOC and solids are supplied internally for each waveband.

These modules were largely rewritten and reorganized to take advantage of the new architecture. In particular, dynamic allocation allowed the expansion of state variables available to the user and the bundling of previously separate modules within the main eutrophication and toxicant modules. Now the user can define an essentially unlimited number of some key state variables. The WASP8 eutrophication module implements the same basic kinetics shown in Figure 13—advanced eutrophication kinetic model for WASP7, but with the following state variables expandable beyond the previous restriction of 3:

- CBOD (5)
- Phytoplankton (10)
- Macroalgae (5)
- Bacteria (5)
- Solids (10)
- Tracer (5)

The WASP8 toxicant module, described more fully by Knightes et al. [54] includes the following state variables as user-selected options (with the expandable limit shown in parentheses):

- Solute chemicals (10)
- Nanochemicals (10)
- Mercury (5)
- Solids (10)
- DOC (5)
- Bacteria (5)
- Tracer (5)
- Salinity
- Temperature

Simulated chemicals can undergo several specified physical and chemical reactions, including equilibrium partitioning, kinetic sorption and desorption, volatilization, photo transformation, hydrolysis, oxidation, reduction and bacterial degradation. A chemical reactant can be linked to multiple chemical and nanochemical products with specified yield coefficients in complex reaction chains. Reaction kinetics are controlled by external forcing functions and chemical-specific reaction coefficients. Both linear reaction constants and nonlinear Monod coefficients can be specified by the user.

Simulated nanochemicals (i.e., nanoparticles) can undergo particle attachment, photo transformation and a general transformation reaction. As with solute chemicals, a nanoparticle can be set to transform into reaction products, which can be solute chemicals or other nanoparticles.

Mercury simulations generally include three components–elemental mercury (Hg^0^), inorganic divalent mercury (HgII) and monomethyl mercury (MeHg) which undergo phase transfer and transformation reactions—Hg^0^ volatilization, Hg^0^ photooxidation, HgII sorption, HgII reduction, HgII photoreduction, HgII methylation, MeHg sorption, MeHg demethylation and MeHg photoreduction. In addition, the user may include two additional mercury state variables representing recalcitrant HgII and MeHg, which are formed by slow sorption or “aging” reactions. This division between labile and recalcitrant (or ‘refractory’) components allows a more accurate simulation of historically contaminated mercury sites.

Simulated bacteria (e.g., ‘microbes’ or ‘pathogens’) may attach to solid particles and settle out of the water column. The simulated population declines with specified death rates for the water and the sediment, salinity toxicity and photolytic mortality for ultraviolet, visible and infrared wavelengths. A simulated bacterial population can include a specified chemical marker which is released upon death. This released chemical is then simulated using the regular solute chemical reactions.

Up to five forms of DOC can be simulated in WASP8. Each may be produced by the dissolution of a simulated biotic solid and each may decay at specified oxidation rates. DOC concentrations contribute to differential light attenuation by wavelength through the water column. DOC forms also complex with solute chemicals, nanochemicals and mercury components, thus altering their reactivity.

Up to ten classes of solids can be simulated in WASP8. For specified size classes of inorganic solids, WASP8 offers mechanistic solids transport options to simulate dynamic settling, deposition, erosion, resuspension, bed load and burial based on a set of parameters and the simulated water column flow velocity. For simple simulations of relatively static water bodies, the user can choose to specify these solid transport velocities directly as spatially variable parameters. Organic solids classes (e.g., plankton, algae, detritus) can also be simulated in WASP8. Production and dissolution rates can be specified with spatially variable parameters and time functions. These time functions can be copied from output of previous eutrophication simulations. The dissolution of biotic solids may yield specified DOC and inert solid fractions. Solids concentrations contribute to differential light attenuation by wavelength through the water column. Solids also provide the medium for sorption and particle attachment of chemicals, nanochemicals, mercury components and bacteria, altering their reactivity.

## 3. The WASP User Community

The WASP user community is made up of professionals from all over the world. WASP is the most widely used continuous simulation water quality model used in the United States and the world. Using web-based analytics we estimate that the install base for WASP is over 15,000 users, mostly located in the United States and Canada. WASP has a large user base in Australia, New Zealand, China, South Korea, Columbia and Peru.

This summer will mark the 21st consecutive year of a Water Quality Modeling Workshop using WASP. The workshop is typically hosted by USEPA’s Region 4 in Atlanta, GA USA and held over a four-day period that includes not only lectures on water quality modeling principles, but also the hands-on development of a real-world model application using WASP. Attendance at these workshops typically numbers between 40 to 75 attendees. This year (2020) the WASP training workshop will be held online. This virtual workshop will cover all the information that is presented at the in-person workshop including hands-on examples. This workshop is offered free of charge and all materials for the workshop made available. There are several universities that use the WASP workshop materials to teach graduate level water quality modeling courses.

WASP workshops have been held in other locations based upon demand and funding. Over the past 20 years a WASP Workshop has been held at eight different EPA Regional offices and six State offices. WASP Workshops have been held internationally in South Korea, China, Russia, Columbia, Peru and Canada.

There is a WASP user community email group that is open to all users. Currently there are more that 100+ users participating in the email group. WASP user support and links to the email support group can be found at [55].

## 4. 50 Years of Model Development; Lessons Learned

The WASP main program was originally written in the FORTRAN language and has remained in that language for its long history, only changing slightly from FORTRAN IV to FORTRAN VII. FORTRAN has stood the test of time and remains one of the most computationally efficient computer languages available even though it is no longer taught at some Universities in their Computer Program/Engineering curriculum. There have been a number of models that originated in FORTRAN but were rewritten to chase the “newest and greatest” language available which in many cases has ultimately resulted in the death of the model.

In many cases computer codes that are most efficient are not necessarily the most useful. WASP is an “engineering” code in that it is well documented, and its logic is intended to be easily understandable and modifiable. The original WASP code [1] was written with the intent that programmers could easily understand and modify the code as required, such as to incorporate new kinetic modules. That design and philosophy has in general been maintained through the history of development of WASP. Other models with which the authors have experience have been initially written for efficiency in a computer programming sense rather than usability in the engineering sense and have been the bane of modelers that have been tasked with using and modifying them, many of which as a result are no longer available.

In contrast, the model interface software (e.g., the graphical user interface) for WASP has evolved from ASCII text card image input files to the present very sophisticated input interface and the post-processing capabilities of the WRDB. These interfaces have evolved as program languages and computer architecture has evolved. One lesson learned though is a caution in tying the model to an existing external software package. At several points during the evolution of WASP this was the case and then that particular software package or system integrated into WASP ceased to exist. That either resulted in WASP remaining static for periods of time or required a rewrite of the supporting models. In addition, for the PC platform WASP and virtually all other software has had to change over time dictated by the changes in the WINDOWS operating systems. If agencies or individuals are interested in the continued support of models, they must be willing to make or support these changes (such as in computer architecture or operating systems) as they occur.

Initially and through WASP5, the source code was distributed with WASP. However, as WASP evolved the code and internal linkages became more complex, the distribution of the source code was limited to model developers. That trend seems common today.

Generalization in the theoretical and computational design of the original WASP model has contributed in large part to its long history of development and use. The basic generalized framework in the present model is in large part the same as that from the 1970s and that described by the first public release in 1981 [1]. However, the WASP model has evolved over the years to incorporate new advances in computer architecture, hydrodynamics and transport and water quality. In addition, WASP has evolved as issues have evolved such as from eutrophication to toxicants and back to eutrophication, harmful algal blooms, organic toxicants (e.g., PCBS, PAHs, etc.) to emerging pollutants and nanoparticles. Many other models that were more specialized in terms of spatial, temporal or kinetic complexity have not stood this test of time. In addition, many models have not responded to changes in the computing environment, such as changes in Microsoft Windows, and have fallen from use.

Another lesson learned is the necessity of providing training and technical support in the use of models such as WASP. The authors have routinely provided WASP workshops for the past 30 or more years. In recent years, that training is provided at USEPA Region 4, with additional specialty workshops being presented on demand around the world. In addition, some online training has been made available using platforms such as YouTube. However, there is no existing sponsored program for either training or technical model support. The authors assist users as time and resources allow and at their discretion. A User group was also created allowing discussions across the large WASP user community.

Another lesson learned is the need for support for model maintenance and development. Water quality models such as WASP provide the vital link between regulating inputs to waterbodies (e.g., point and non-point source loads) and water quality targets (e.g., standards and criteria). However, in the U.S., with few exceptions there is a lack of established and funded programs that provide consistent and meaningful support for the development and maintenance of water quality models, for training in their use and for user technical support. Since the costs of mistakes in environmental decision making are often large, it would seem cost effective to ensure that decisions are made using the best information and models available applied by users that are adequately trained and supported in their use.

## 5. Future Development

It is difficult to determine future enhancements to WASP because of uncertainties around funding and available development time. Nevertheless, we anticipate work in several areas.

First, we respond to requests from the WASP user community to address specific needs, which tend to drive innovation and improvement in the scientific models. Ease of use is also a key consideration and user interface enhancements are often the most immediately apparent improvements.

Next, we continually look for ways to improve the runtime performance of the model. Since the 1980s, computing capabilities have steadily become faster and more robust. We endeavor to improve WASP by exploiting these ever-increasing computing capabilities. This allows users to construct larger and more detailed model networks and simulate longer periods of time.

We have recently begun to develop WASPTool, a set of software utilities to communicate with the WASP GUI and scientific modules using external programs or methods. This will make WASP more interoperable with other software in an ever-changing computer environment.

Lastly, the yearly WASP training workshop will be made available online. This virtual workshop will cover all the information that is presented at the in-person workshop including hands-on examples.
